# Anthracycline-free tumor elimination in mice leads to functional and molecular cardiac recovery from cancer-induced alterations in contrast to long-lasting doxorubicin treatment effects

**DOI:** 10.1007/s00395-021-00902-7

**Published:** 2021-10-20

**Authors:** Stefan Pietzsch, Katharina Wohlan, James T. Thackeray, Maren Heimerl, Sven Schuchardt, Michaela Scherr, Melanie Ricke-Hoch, Denise Hilfiker-Kleiner

**Affiliations:** 1 Department of Cardiology and Angiology, Hannover Medical School, Carl-Neuberg Str. 1, 30625 Hannover, Germany; 2 Department of Molecular and Cellular Biology, Baylor College of Medicine, Houston, USA; 3 Department of Nuclear Medicine, Hannover Medical School, Hannover, Germany; 4 Fraunhofer Institute for Toxicology and Experimental Medicine ITEM, Hannover, Germany; 5 Department of Hematology, Hemostasis, Oncology and Stem Cell Transplantation, Hannover Medical School, Hannover, Germany; 6 Department of Cardiovascular Complications of Oncologic Therapies, Medical Faculty of the Philipps University Marburg, Marburg, Germany

**Keywords:** Cardio-oncology, Cancer, Heart, Regeneration

## Abstract

Systemic effects of advanced cancer impact on the heart leading to cardiac atrophy and functional impairment. Using a murine melanoma cancer model (B16F10 melanoma cells stably transduced with a Ganciclovir (GCV)-inducible suicide gene), the present study analysed the recovery potential of cancer-induced cardiomyopathy with or without use of doxorubicin (Dox). After Dox-free tumor elimination and recovery for 70 ± 5 days, cancer-induced morphologic, functional, metabolic and molecular changes were largely reversible in mice previously bearing tumors. Moreover, grip strength and cardiac response to angiotensin II-induced high blood pressure were comparable with healthy control mice. In turn, addition of Dox (12 mg/kg BW) to melanoma-bearing mice reduced survival in the acute phase compared to GCV-alone induced recovery, while long-term effects on cardiac morphologic and functional recovery were similar. However, Dox treatment was associated with permanent changes in the cardiac gene expression pattern, especially the circadian rhythm pathway associated with the DNA damage repair system. Thus, the heart can recover from cancer-induced damage after chemotherapy-free tumor elimination. In contrast, treatment with the cardiotoxic drug Dox induces, besides well-known adverse acute effects, long-term subclinical changes in the heart, especially of circadian clock genes. Since the circadian clock is known to impact on cardiac repair mechanisms, these changes may render the heart more sensitive to additional stress during lifetime, which, at least in part, could contribute to late cardiac toxicity.

**Supplementary Information:**

The online version contains supplementary material available at 10.1007/s00395-021-00902-7.

## Introduction

Cancer and heart disease are major causes of death in western countries [46]. Accumulating evidence suggests multiple pathophysiological interactions between cancer, anti-cancer therapies and heart failure [9, 41]. While cardiotoxic effects of anti-cancer therapies have been in focus for decades, the effects of cancer itself on the heart are less known. Among direct effects of cancer on the heart is cardiac atrophy in advanced cancer stages [5, 29, 40], caused by cardiomyocyte cell size reduction and/or cardiomyocyte loss [8, 27, 41]. Cardiac atrophy and functional loss are partly caused by an imbalance of cardiac protein synthesis and degradation processes, in part due to impairment in systemic and cardiac metabolism involving deregulated insulin signaling [29, 43] and cancer metabolites [21, 42]. Among cardiotoxic agents, anthracyclines (AC) such as Dox are highly efficient anti-cancer drugs whose usage is somewhat limited by their cardiotoxic effects [47]. Early anthracycline-induced cardiotoxicity can be detected at various levels of severity during treatment and within the first 12 months after completing AC chemotherapy. Acute myocardial injury documented by cardiac imaging and high sensitivity troponin assays appears in 50% of AC receivers [31]. Late AC-induced cardiotoxicity culminates in heart failure years or even decades after AC chemotherapy [15]. Late AC-induced cardiotoxicity is a growing clinical problem, accounting for more than 50% of premature death in survivors of pediatric cancer who received AC [15]. Anthracycline-induced cardiotoxicity represents a continuous phenomenon that includes increased oxidative stress, poisoning of topoisomerase 2b, DNA damage, mitochondrial and myocardial cell injury leading to progressive heart failure, that can be enhanced by additional cardiac stress stimuli such as extensive physical activity, pregnancy and untreated high blood pressure later in life [6, 33]. Finally, both cancer disease and anti-cancer treatment induce alterations in signaling pathways and gene expression in the heart [22, 34, 39, 43]. The simultaneous impact of both conditions in the heart is barely studied since most experimental models study either cancer-induced effects or cancer therapy-induced damage separately.

In the present study, we aimed to distinguish cancer-induced myocardial damage from Dox-induced cardiotoxic side effects in a preclinical mouse model of melanoma disease. Therefore, we generated a reversible melanoma tumor model in mice (B16F10 melanoma cells transduced with herpes simplex virus type 1 thymidine kinase (HSVtk), B16F10HSVtk-c [35]), in which severe cancer-induced cardiac damage could be initiated and cancer could be subsequently eliminated without AC chemotherapy [44]. In this model, we observed that advanced cancer-induced cardiomyopathy with massive atrophy and functional loss was nearly completely reversible at the functional, morphological, metabolic and molecular level. Moreover, after recovery from cancer, mice displayed normal physiological (weight lifting) and pathophysiological (angiotensin II (AngII)-induced high blood pressure) stress tolerance at follow-up. In contrast, after confirming Dox sensitivity of the B16F10 melanoma cell line, the same model combined with a moderate cumulative dose of Dox (12 mg/kg BW) treatment led to high mortality. Even though surviving mice displayed functional and morphological cardiac recovery, long-lasting changes in the cardiac gene expression pattern with prominent changes in the circadian rhythm pathway were evident indicating that rather Dox treatment than the cancer itself might be responsible for late-onset cardiotoxicity after cancer disease.

## Materials and methods

### Cell line and cell culture experiments

#### B16F10 melanoma cells

Murine melanoma cell line B16F10 was obtained from ATCC. Cells were grown in DMEM culture medium containing 4.5 g/L glucose (Biochrom AG) supplemented with 10% FCS (Biochrom AG) and penicillin/streptomycin (100 U/100 µg per ml, Gibco). For injection, tumor cells were grown to confluence and detached from cell culture flasks by treatment with 0.25% trypsin/EDTA (Gibco). Trypsin reaction was arrested by addition of culture medium, and cell suspension was centrifuged. Cell pellet was washed twice with prewarmed, sterile PBS; then, cells were suspended in PBS and counted.

B16F10 cells were genetically modified by lentiviral transduction with a herpes simplex virus type 1 thymidine kinase (HSVtk), firefly luciferase and yellow fluorescent protein (YFP) containing construct [35]. Single cell clones were isolated by limiting dilution and checked for positive YFP signal in FACS. A fully YFP-positive cell clone (B16F10HSVtk-c) was used for all in vitro and in vivo experiments. HSVtk1 enabling cell-specific induction of apoptosis was activated by addition of ganciclovir as described [44]. Sensitivity to Dox treatment has been tested in vitro in B16F10-HSVtk cells by treating the cells with 0.05 and 0.25 μg/μl Dox compared to vehicle-treated cells for 72 h.

#### Experiments with neonatal rat cardiomyocytes (NRCM)

Isolation of NRCM and cultivation in DMEM high glucose 4.5 g/L and M199 (4:1) was similar performed as described [13]. In brief, NRCM were sacrificed by decapitation and hearts were harvested. Cardiomyocytes were isolated by collagenase digestion followed by purification using Percoll gradient centrifugation [30].

si-RNA-mediated knockdown of *Bmal1* was performed by transfection of a pool of four specific siRNAs (Dharmacon Horizon) at a final concentration of 50 nM using Dharmafect-1 (Dharmacon Research Inc) transfection reagent in accordance to the manufacturer’s instructions and analyses with regard to Dox stimulation were carried out 48 h after transfection. In case of Dox stimulation, NRCM were incubated with 0.2 µg/ml for 24 h followed by protein isolation.

#### TMRE assay of NRCM

After si-RNA-mediated knockdown of *Bmal1* and controls (48 h) followed by 24 h Dox stimulation, the mitochondrial membrane potential was measured in NRCM using the TMRE mitochondrial membrane potential assay kit (abcam, ab113852), according to the manufacturer’s protocols. In brief, 20 μM FCCP was added to the medium of NRCM FCCP control cells 10 min prior to TMRE staining. NRCM were incubated with 500 nM TMRE for 20 min. Afterwards, media were removed and cells were washed twice with PBS/0.2% BSA. The mitochondrial membrane potential was measured with a fluorescence plate reader (Thermo Scientific Varioskan Flash; SkanIt Software 2.4.5) at Ex/Em 549/575 nm and fluorescence microscopy (Axio Observer 7 and Zen 2.6 pro software, Carl Zeiss).

#### Protein fractionation of the cytosol of NRCM

After si-RNA-mediated knockdown of *Bmal1* and controls (48 h) followed by 24 h Dox stimulation, the cytosolic protein fraction was isolated using a subcellular protein fractionation kit for cultured cells (Thermo Scientific 78,840) according to the manufacturer’s protocols followed by SDS-PAGE protein expression analysis (as described below in section protein analyses and western blotting).

#### In situ cell death detection assay (TUNEL)

After si-RNA-mediated knockdown of *Bmal1* and controls (48 h) followed by 24 h Dox stimulation, apoptotic NRCM were fixed and identified with the in situ cell death detection kit, fluorescein (Roche, 11684795910) according to the manufacturer’s protocols and fluorescence microscopy using an Axio Observer 7 and Zen 2.6 pro software (Carl Zeiss). Nuclear staining was performed with DAPI Hoechst 33342 (SIGMA-Aldrich).

### Animal experiments

#### Melanoma model

Male mice (12 ± 2 weeks of age) were injected intraperitoneally (i.p.) with B16F10HSVtk-c melanoma cells (HSVtk-m, 1 × 10^6^) or PBS (PBS-m) as a vehicle as described [43].

Treatment with ganciclovir (GCV, 2 × 80 mg/kg BW/day) was started at advanced tumor disease stage based on the guidelines of recognition of distress in experimental animals proposed by Morton and Griffith [28]. GCV treatment of control mice (GCV-m) was started in parallel. GCV treatment was continued until signal loss in the in vivo imaging system (IVIS) occurred (Rec-m). For live visualization of melanoma cells in IVIS, mice were imaged using an IVIS Lumina II (Caliper Life Sciences) for 1 min following i.p. injection of 100 μl of 8 μg/μl D-Luciferin (AppliChem). Living Image 3.1 software was used to analyse bioluminescence intensity. After melanoma cell implantation, HSVtk-m and PBS-m mice received continuous analgesia (Novalgin, 1000 mg/kg/day in drinking water). Food intake in mice with advanced B16F10 cancer disease and at the time of randomization into the several experimental groups was controlled as previously described [43]. Mice were housed in groups of five and maintained on a 14 h/10 h light/dark cycle with standard laboratory chow and water freely available. Animal health condition was assessed based on the guidelines of recognition of distress in experimental animals as proposed by Morton and Griffith [28]. All animal studies were conducted in accordance with German animal protection law and with the European Communities Council Directive 86/609/EEC and 2010/63/EU for the protection of animals used for experimental purposes. All experiments were approved by the local institutional animal care and research advisory committee and permitted by LAVES (Niedersächsisches Landesamt für Verbraucherschutz und Lebensmittelsicherheit; Oldenburg, Lower Saxony, Germany).

#### Angiotensin II treatment

AngII treatment was started after melanoma cell signal loss in IVIS in Rec-m (Rec-m+AngII) and tumor-free GCV-m served as control (GCV-m + AngII). Via osmotic mini pumps (Alzet^®^) AngII was applied for 14 days at a daily dose of 1.44 µg/g BW.

#### Doxorubicin treatment

Single doses of 4 mg/kg BW of doxorubicin treatment were applied i.p. at day 7, 10 and 13 after tumor cell injection. Treatment was completed with GCV (daily dosage above) until signal loss in IVIS (Rec-m + Dox).

#### Grip strength assessment

Grip strength was assessed as previously described [10]. In brief, weights were mounted on an easy to grab steel sponge and mice had to hold on to the sponge for at least 3 s to reach the next round. Each round 5 g of weight was added until mice failed at 3 tries to hold the weights for at least 3 s. Individual GS score was calculated including maximum held weight and holding time at failure normalized on individual body weights, respectively, tibia length for the Rec-m + Dox mice.

#### Echocardiography

Echocardiography was performed of anesthetized mice as described previously [43].

In brief, contractile function and heart rate were assessed by serial echocardiography using the Vevo 2100 system (VisualSonics) at baseline (before tumor cell injection), at advanced disease states (day 18 ± 2 after tumor cell injection) and after recovery (day 70 ± 5 after tumor cell injections). Anesthesia was induced with 4% (in 100% oxygen) isoflurane, followed by maintenance at 0.5–1% isoflurane via a special vaporizer for rodents delivered by a small nose cone (VisualSonics). For echocardiographic image acquisition, the animal was placed in a supine position on a prewarmed platform and the body temperature was maintained as close to 37 °C as possible during the entire procedure. Echocardiographic measurements were obtained 5 min following the induction of anesthetic when heart rate had stably recovered to exclude the variation in cardiac function created by time after induction. Parasternal short- or long-axis views were recorded in B-mode at the level of the papillary muscle, and still images were used to measure LV end-diastolic diameter (LVEDD) and LV end-systolic diameter (LVESD) to calculate fractional shortening ([LVEDD–LVESD]/LVEDD × 100) and fractional area change ([end-diastolic area − end-systolic area]/end-diastolic area × 100). Cardiac output, LV stroke volume, diastolic endocardial volume, and endocardial systolic volume were calculated by VisualSonics Vevo 2100 software version 3.

#### FDG-PET-CT

^18^F-FDG-PET-CT images were acquired in anesthetized mice as described in [43]. Cardiac uptake was analysed semi-quantitatively in manually defined regions of interest as percent injected dose (ID)/g tissue at 50–60 min after tracer administration.

#### Organ/tissue harvesting

Mice were euthanized (cervical dislocation) and hearts were harvested, weighed, and halves were directly frozen for subsequent analyses and stored at − 80 °C.

### Histology and immunostaining

For cardiac morphological analyses, hearts were embedded in OCT and frozen at − 80 °C.

Subsequently, cryosections were stained with hematoxylin and eosin (H&E) or wheat germ agglutinin (WGA) as described previously [16]. Cross-sectional area was determined on longitudinal cryosections after wheat germ agglutinin/Hoechst staining. At least 30 cardiomyocytes per heart were counted.

### RNA isolation and qRT-PCR

Total RNA from adult murine hearts or skeletal muscle (SKM) was isolated with Trizol (Invitrogen), and cDNA synthesis was performed as described previously [14, 18]. Real-time PCR with SYBR green dye method (Brilliant SYBR Green Mastermix-Kit, Thermo Fisher) was performed with the AriaMx Real-Time PCR System (Agilent Technologies) as described in [43]. Semi-quantitative analyses were based on normalization to 18S or beta-2-microglobulin (B2M) expression. The list of qRT-PCR primers used in this study is provided below.

### Sequences of qRT-PCR primers

18S rRNA (sense: GTAACCCGTTGAACCCCATT, antisense: CCATCCAATCGGTAGTAGCG), *Atrogin1* (sense: CTTCTCGACTGCCATCCTGG, antisense: GTTCTTTTGGGCGATGCCAC), *Bmal1* (sense: TGCCCTCTGGAGAAGGTGG, antisense: GGGAGGCGTACTTGTGATGT), *B2M* (sense: CATGGCTCGCTCGGTGACC, antisense: AATGTGAGGCGGGTGGAACTG), *Ciart* (sense: GGTCGCTTTGACAGAGGATT, antisense: GCTGTGTTTGGCCCATCTTC), *CLOCK* (sense: GCCTCAGGCACGTGAAAGAA, antisense: GGAGTCTCCAACACCCACAG), *Cry1* (sense: GCATCAACAGGTGGCGATTTT, antisense: TGTGAAATGCGCACGATGAC), *Cry2* (sense: GCATCAACCGATGGAGGTTC, antisense: TCCCGTTCTTTCCCAAAGGG), *Myh7* (sense: CAAGTTCCGCAAGGTGC, antisense: AAATTGCTTTATTCTGCTTCCAC), *Irs2* (sense: CCATCGATGTGAGAGGCGAG, antisense: GGCGATGGGGCTGGTAG), *Gadd45a* (sense: CTGCAGAGCAGAAGACCGAA, antisense: ATGAATGTGGGTTCGTCACCA), *Mbnl2* (sense: CGACACAAACGACAACACCG, antisense: ATGGGTACTGTGGGGATGAAC), *Hnrnpa1* (sense: ACGGAACCAAGGTGGCTATG, antisense: TGTGCTTGGCTGAGTTCACA), *Per2* (sense: CGCGGATATGTTTGCTGTGG, antisense: GGAGGGATTCTAGGCGCTTC).

### RNA-Seq.

Total RNA from adult murine left ventricles was isolated with Trizol (Invitrogen) and *n* = 2–4 individuals were pooled resulting in 3 pool samples per group (for: PBS-m, GCV-m, HSVtk-m, Rec-m and Rec-m + Dox). RNA-Seq. was performed as described previously [17, 18]. RNA integrity/quality and quantification were controlled by bioanalyzer RNA Analysis (Agilent).

#### Library generation, quality control, and quantification

500 ng of total RNA per sample were utilized as input for mRNA enrichment procedure with ‘NEBNext^®^ Poly(A) mRNA Magnetic Isolation Module’ (E7490L; New England Biolabs) followed by stranded cDNA library generation using ‘NEBNext^®^ Ultra II Directional RNA Library Prep Kit for Illumina’ (E7760L; New England Biolabs). All steps were performed as recommended in user manualE7760 (Version 1.0_02-2017; NEB) except that all reactions were downscaled to 2/3 of initial volumes. Furthermore, one additional purification step was introduced at the end of the standard procedure, using 1× ‘Agencourt^®^ AMPure^®^ XP Beads’ (#A63881; Beckman Coulter, Inc.).

cDNA libraries were barcoded by dual indexing approach, using ‘NEBNext Multiplex Oligos for Illumina–96 Unique Dual Index Primer Pairs’ (6440S; New England Biolabs). All generated cDNA libraries were amplified with nine cycles of final PCR.

Fragment length distribution of individual libraries was monitored using ‘Bioanalyzer High Sensitivity DNA Assay’ (5067-4626; Agilent Technologies). Quantification of libraries was performed by use of the ‘Qubit^®^ dsDNA HS Assay Kit’ (Q32854; ThermoFisher Scientific).

#### Library denaturation and sequencing run

Equal molar amounts of fifteen individually barcoded libraries were pooled. Accordingly, each analysed library constitutes 6.7% of overall flowcell/run capacity. The library pool was denatured with NaOH and was finally diluted to 1.8 pM according to the Denature and Dilute Libraries Guide (Document # 15048776 v02; Illumina). 1.3 ml of denatured pool were loaded on an Illumina NextSeq 550 sequencer. Two subsequent runs were performed and respective data were combined. Accordingly, two High Output Flowcells for 75 bp single reads (20024906; Illumina) were used in total. Sequencing was performed with the following settings: Sequence read 1 with 76 bases; Index reads 1 and 2 with 8 bases each.

#### BCL to FASTQ conversion

BCL files were converted to FASTQ files using bcl2fastq Conversion Software version v2.20.0.422 (Illumina).

#### Raw data processing and quality control

Raw data processing was conducted by use of nfcore/rnaseq (version 1.3) which is a bioinformatics best-practice analysis pipeline used for RNA sequencing data at the National Genomics Infrastructure at SciLifeLab Stockholm, Sweden. The pipeline uses Nextflow, a bioinformatics workflow tool. It pre-processes raw data from FastQ inputs, aligns the reads and performs extensive quality control on the results. The genome reference and annotation data were taken from GENCODE.org (Mus musculus; GRCm38; release M18).

#### Normalization and differential expression analysis (without outlier filtering)

Normalization and differential expression analysis were performed with DESeq2 (Galaxy Tool Version 2.11.40.2) with default settings except for “Output normalized counts table”, “Turn off outliers filtering”, and “Turn off independent filtering”, all of which were set to “True”.

#### Data analysis

Pathway analysis was performed using the software DAVID Bioinformatics Resources 6.8 [19, 20]. Therefore, pre-filtered RNA-Seq. data gene list (filter criteria: base mean read count of ≥ 100 and an adjusted *P* value ≤ 0.01 or ≤ 0.05 depending on the respective comparison as described in the tables) was analysed with the functional annotation tool based on KEGG pathways.

### Protein analyses and Western blotting

Protein expression levels were determined by Western blotting, using SDS PAGE as previously described [43]. The following primary and secondary antibodies were used:

pAKT (S473) Cell Signaling Technology (CST) #4060; AKT, CST #9272; Bmal1 CST #14020, Caspase 3, CST #9662; CD36 abcam ab80080; Cytochrome C, CST #11940; GAPDH, CST #2118; phospho-H2AX (Ser139), CST #9718; PARP CST #9542; donkey anti-Rabbit IgG, peroxidase-linked species-specific whole antibody NA934V (GE Healthcare) and rat IgG HRP-conjugated antibody HAF005 (R&D Systems).

### Insulin and metabolomics measurement

Murine plasma samples were acquired by centrifugation of right ventricular blood in EDTA containing vials and stored at − 80 °C. Insulin concentration in plasma was determined using a commercial ELISA kit (EZRMI-13 K Rat/Mouse Insulin, Merck Millipore) following the manufacturer’s protocol. AbsoluteIDQ™ p180 Kit (BIOCRATES Life Sciences AG) was used for metabolomic analyses and was kindly provided by BIOCRATES Life Sciences AG.

### Statistical analyses

Database management and statistical analyses were performed with the PRISM version 5.0 or 7.0 statistical programme (GraphPad Software Inc., La Jolla, CA, USA). Data are presented as mean ± SD. Differences between groups were analysed by Student’s *t* test with or without Welch's correction or ANOVA with Bonferroni post hoc tests. A non-parametric Mann–Whitney test was performed on data not fitting a Gaussian distribution (as analysed by D’Agostino and Pearson omnibus normality test or Shapiro–Wilk normality test if the sample size was too small for D’Agostino and Pearson omnibus normality test.). A two-tailed *P* value of < 0.05 was considered statistically significant.

## Results

### Generation of an HSVtk-luciferase melanoma cancer model in mice

**Fig. 1:**
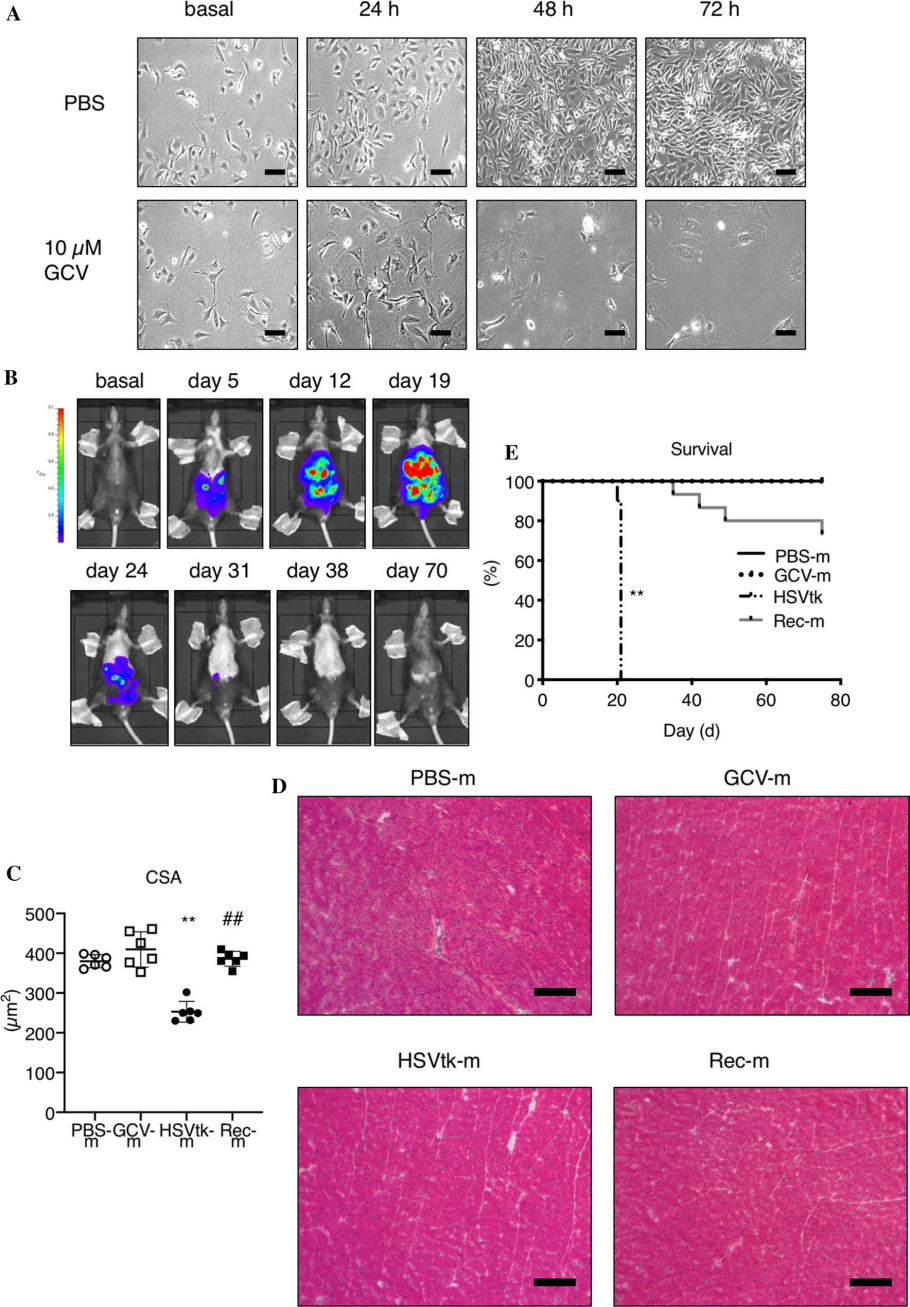
Establishing a melanoma cancer model in mice enabling cancer cell specific and anthracycline-free tumor elimination. **A** B16F10 melanoma cells stably expressing herpes simplex thymidine kinase and luciferase (B16F10HSVtk-c) were treated with Ganciclovir (GCV, 10 µM) for 72 h in vitro. **B** Exemplary images from male C57BL6/N injected with B16F10HSVtk-c; tumor growth was observed via in vivo imaging system (IVIS). Mice were treated with ganciclovir (GCV) starting around day 16 after B16F10HSVtk-c injection and treatment was continued until signal loss in IVIS. **C**, **D** Cardiomyocyte cross-sectional area (CSA) after WGA staining and hematoxilin/eosin staining in left ventricular cryosections in age-matched controls without cancer cell injection and without GCV treatment (PBS-m, *n* = 6), healthy age-matched controls without cancer cell injection and with GCV treatment (GCV-m, *n* = 6), tumor-bearing mice with advanced tumor burden (HSVtk-m, *n* = 6), mice recovered from B16F10HSVtk-c-induced tumor burden after GCV treatment (Rec-m, *n* = 6) and mice recovered from B16F10HSVtk-c-induced tumor burden after GCV treatment. **E** Survival of mice after injection with B16F10HSVtk-c with (Rec-m, *n* = 15) or without (HSVtk-m, *n* = 10) GCV treatment compared to non-tumor mice with PBS injection instead of cancer cells with (GCV-m, *n* = 7) or without (PBS-m, *n* = 15) GCV treatment. Data are depicted as mean ± SD, **P < 0.01 vs. respective non-tumor group and ^##^*P* < 0.01 vs. respective non-GCV group using Log-rank Mantel-Cox test or two-way ANOVA followed by Bonferroni post hoc tests as required

Clonally expanded B16F10 cells stably expressing the HSVtk death receptor and luciferase (B16F10HSVtk-c) were tested in culture for GCV-induced cell death (Fig. 1A). Subsequently, 1 × 10^6 cells were injected i.p. in male mice (10–12 weeks of age, HSVtk-m) and tumor growth was monitored by IVIS (Fig. 1B). At advanced tumor stage, mice presented a significant loss in body weight (BW), gastrocnemius weight (GW), soleus weight (SW) and heart weight (HW) associated with reduced cardiomyocyte size, left ventricular (LV) function, dimension and output compared to healthy controls (Table 1, Fig. 1C) but no signs of substantial structural remodeling in LV tissue, which would indicate enhanced inflammation or fibrosis (Fig. 1D).

**Table 1:** Morphology and echocardiography

	PBS-m	GCV-m	HSVtk-m	Rec-m
**Weights**	*n* = 15	*n* = 7	*n* = 9	*n* = 8
BW (g)	32 ± 2.6	32.2 ± 3.1	25.6 ± 1.2**	28.1 ± 1.1**
HW (g)	123.6 ± 4.4	116.1 ± 10.2	83.6 ± 4.6**	109.0 ± 14.2^##^
HW/TL (mg/mm)	7.3 ± 0.2	6.8 ± 0.6	5.0 ± 0.3**	6.5 ± 0.8^##^
GW/TL (mg/mm)	9.5 ± 0.7	9.8 ± 0.5	6.3 ± 0.70**	8.9 ± 0.8^##^
SW/TL (mg/mm)	0.57 ± 0.07	0.59 ± 0.07	0.37 ± 0.04**	0.54 ± 0.06^##^
**Echocardiography**	*n* = 15	*n* = 7	*n* = 25	*n* = 9
FS (%)	32.1 ± 3.5	38.3 ± 1.3	25.5 ± 5.4**	35.3 ± 2.7^##^
LVEDD (mm)	7.4 ± 0.3	7.6 ± 0.4	6.7 ± 0.5**	7.4 ± 0.3^#^
LVESD (mm)	6.2 ± 0.4	6.1 ± 0.4	5.8 ± 0.5**	6.2 ± 0.3
HR (bpm)	572 ± 34	599 ± 23	473 ± 77**	587 ± 27^##^
CO (ml/min)	19.6 ± 2.5	20 ± 2.3	11.2 ± 3.3**	20.4 ± 2.1^##^
ESV (µl)	33.7 ± 4.7	33.5 ± 3.9	23.7 ± 6.9**	34.3 ± 3.2^##^
EVd (µl)	58.2 ± 7.0	60.2 ± 8.4	45.3 ± 12.4**	64.3 ± 7.4^##^
EVs (µl)	27.2 ± 4.7	26.6 ± 5.7	18.3 ± 9**	30.0 ± 5.0^##^

Data are depicted as mean ± SD, **P < 0.01 vs. respective non-tumor group and ^#^*P* < 0.05, ^##^*P* < 0.01 vs. respective non-GCV group, using two-way ANOVA followed by Bonferroni post hoc tests

*BW* Body weight, *HW* heart weight, *TL* tibia length, *GW* gastrocnemius weight, *SW* soleus weight, *FS* fractional shortening, *LVEDD* left ventricular end-diastolic diameter, *LVESD* left ventricular end-systolic diameter, *HR* heart rate, *CO* cardiac output, *ESV* endocardial stroke volume, *EVd* endocardial volume diastolic, *EVs* endocardial volume systolic, from untreated (PBS-m) and GCV-treated (GCV-m) tumor-free control mice, tumor-bearing (HSVtk-m) and GCV-treated and recovered (Rec-m) mice

### Cardiac function, dimension and output after GCV-induced tumor elimination in mice with advanced melanoma cancer

At advanced tumor disease stage of HSVtk-m (around day 16 after tumor cell inoculation), mice (*n* = 25) were randomized to groups with (*n* = 15) or without (*n* = 10) GCV treatment. GCV treatment was continued until complete tumor elimination after 35 ± 5 days as indicated by loss of IVIS signal (Rec-m, Fig. 1B) followed by a recovery period until day 70 ± 5. Latest by 21 days post-tumor induction, all untreated HSVtk-m had died or were sacrificed for animal welfare reasons. GCV-treatment in the treatment group of tumor bearing mice (Rec-m) was needed until day 35 ± 5 to result in complete loss of tumor signal in IVIS. Despite loss of tumor signal, 46% of Rec-m experienced relapse of tumor disease and 26% died prior to day 75 (Fig. 1E). The surviving Rec-m without relapse were sacrificed at 70 ± 5 days after tumor injection and compared to age-matched control mice without cancer disease treated with PBS (PBS-m) or PBS and GCV (GCV-m). Further analyses showed that Rec-m displayed similar GW, SW, HW, LV tissue morphology, cardiomyocyte size, cardiac function and dimension when compared to control PBS-m or GCV-m (Table 1, Fig. 1C–E).

### Metabolic analyses revealed normalization of cardiac metabolic marker shifts, metabolites and tumor markers after recovery from cancer

**Fig. 2:**
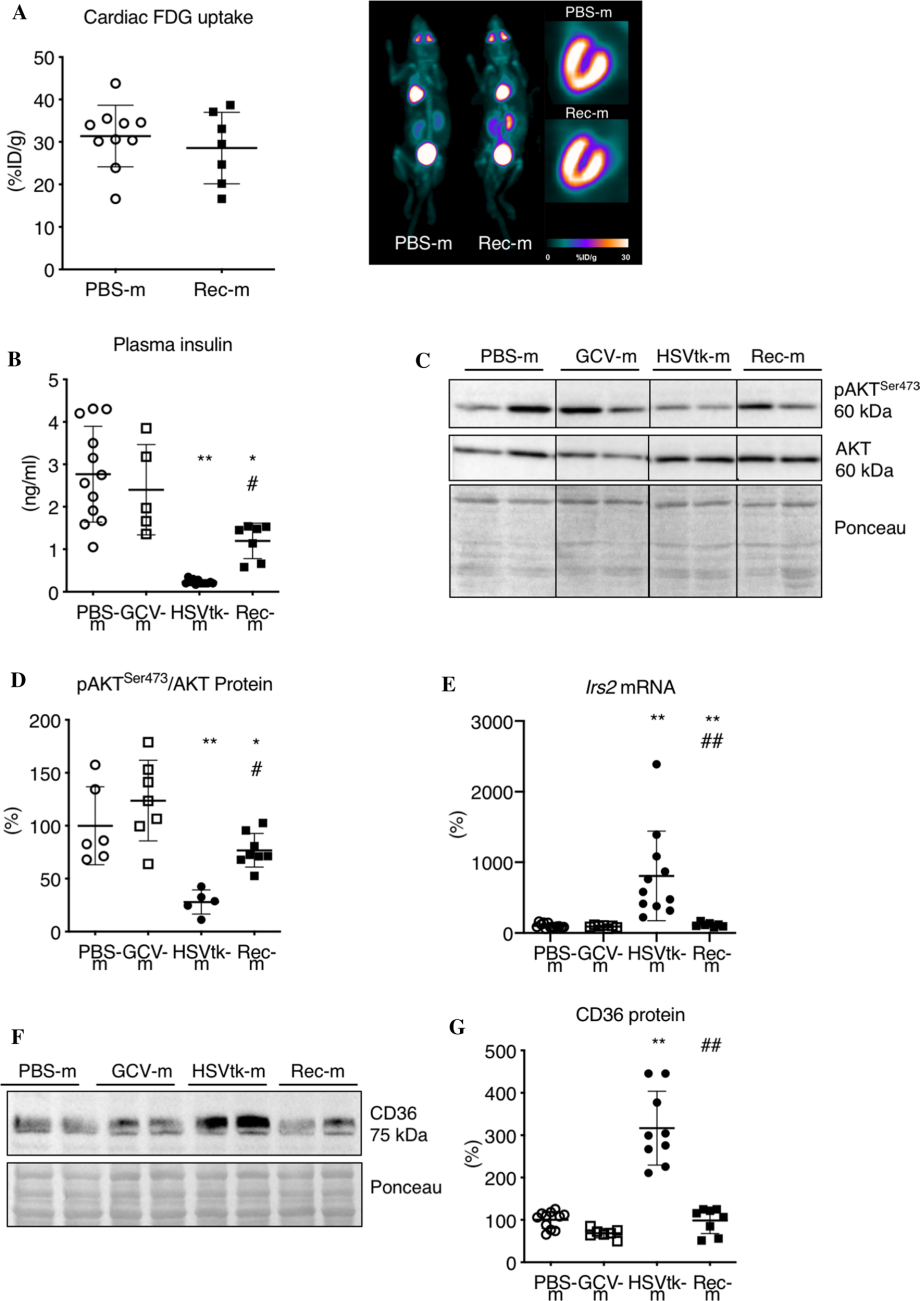
Cardiac metabolic phenotype after anthracycline-free tumor elimination and recovery from cancer. **A** Representative whole body and long-axis myocardial ^18^F-FDG PET/CT images, with graph summarizing quantitative uptake (percentage injected dose per gram tissue [%ID/g]) from healthy control mice (PBS-m, *n* = 9) and mice recovered from B16F10HSVtk-c induced tumor burden after GCV treatment (Rec-m, *n* = 7). **B** Determination of plasma insulin levels in PBS-m (*n* = 12), GCV-m (*n* = 5), HSVtk-m (*n* = 12) and Rec-m (*n* = 7). **C**, **D** Representative western blot images of phospho-(Ser473) and total AKT from LV tissue of PBS-m (*n* = 6), GCV-m (*n* = 7), HSVtk-m (*n* = 5) and Rec-m (*n* = 8) and corresponding quantification normalized on Ponceau staining. **E** Quantitative mRNA levels (qRT-PCR) of *Irs2* in LV tissue from PBS-m (*n* = 13), GCV-m (*n* = 7), HSVtk-m (*n* = 11) and Rec-m (*n* = 7) normalized to 18S. **F**, **G** Representative western blot images of CD36 from LV tissue of PBS-m (*n* = 11), GCV-m (*n* = 7), HSVtk-m (*n* = 9) and Rec-m (*n* = 8) and corresponding quantification normalized on Ponceau staining. Data are depicted as mean ± SD, *P < 0.05, **P < 0.01 vs. respective non-tumor group and ^#^*P* < 0.05, ^##^*P* < 0.01 vs. respective non-GCV group using 2-way ANOVA followed by Bonferroni post hoc tests or two-tailed Student’s t test as required

We previously showed that advanced tumor disease reduced cardiac glucose uptake more than 60% and also affected insulin signaling [43]. After GCV-induced tumor cell elimination and recovery, cardiac glucose uptake (PET-CT, ^18^F-FDG-uptake) in Rec-m was comparable to PBS-m (Fig. 2A). In addition, reduced plasma insulin levels at advanced tumor stage had significantly improved in Rec-m, albeit not comparable to levels of GCV-m (Fig. 2B). Despite lower plasma insulin levels, Rec-m showed complete normalization of cardiac insulin signaling indicated by increased cardiac AKT phosphorylation and reduced *Irs2* expression (Fig. 2B-E, Suppl. Fig. 1A–C). In the same line, reduced GLUT4 and increased GLUT1 mRNA expression in HSVtk-m had normalized in Rec-m (Suppl. Fig. 2A, B). Metabolic analyses revealed a significant reduction of hexoses, carnitine, arginine, citrulline and methionine plasma levels while plasma levels of glutamic acid and sarcosine were increased in HSVtk-m, which all normalized to levels of healthy PBS-m and GCV-m in Rec-m (Table 2). Advanced tumor disease was associated with an upregulation of the fatty acid transporter CD36 in LV tissue in HSVtk-m, which was also normalized in Rec-m (Fig. 2F, G, Suppl. Fig. 1D, E).

**Table 2:** Metabolic analyses in recovered mice

(µM)	PBS-m (*n* = 10)	GCV-m (*n* = 5)	HSVtk-m (*n* = 12)	Rec-m (*n* = 6)
Hexoses	9861 ± 1516	10,920 ± 2189	5842 ± 2101**	9578 ± 1427^##^
Carnitine	18.8 ± 3.4	19.9 ± 1.5	13.2 ± 3**	22.6 ± 2.4^##^
Arginine	124.1 ± 20.6	105.7 ± 24	48.2 ± 29.4**	98.2 ± 6.7^##^
Citrulline	59 ± 13.1	68.1 ± 1 1.1	43.6 ± 9.1**	62.1 ± 6.3^##^
Glutamic acid	73.1 ± 25.5	58.7 ± 11	146.6 ± 67.7**	61.8 ± 24.4^##^
Methionine	49.1 ± 9.6	46.6 ± 6.9	35.5 ± 10.5**	46.3 ± 5.6^#^
Sarcosine	16 ± 3.7	16.3 ± 5.9	27.9 ± 10.7**	18.3 ± 8.3^#^
LysoPC C18:0	72.7 ± 8.6	72.8 ± 10.2	98.4 ± 17.5**	64.6 ± 11.2^##^
LysoPC C18:1	36.9 ± 5	44.6 ± 5.7	27.7 ± 7**	41.1 ± 7.9^##^
SM C16:0	14.9 ± 1.5	14.9 ± 2.2	29.5 ± 7.3**	14.5 ± 3.4^##^
SM C24:1	8.3 ± 2.9	7.3 ± 1.3	15.8 ± 3.7**	9.4 ± 1.8^##^

Metabolic parameters from untreated (PBS-m) and GCV-treated (GCV-m) tumor-free control mice, tumor-bearing (HSVtk-m) and GCV-treated and recovered (Rec-m) mice. Data are depicted as mean ± SD, **P < 0.01 vs. respective non-tumor group and ^#^*P* < 0.05, ^##^*P* < 0.01 vs. respective non-GCV group, using two-way ANOVA followed by Bonferroni post hoc tests

*LysoPC* lysophosphatidylcholine, *SM* sphingomyelin

### Physical strength in mice recovered from advanced cancer disease

**Fig. 3:**
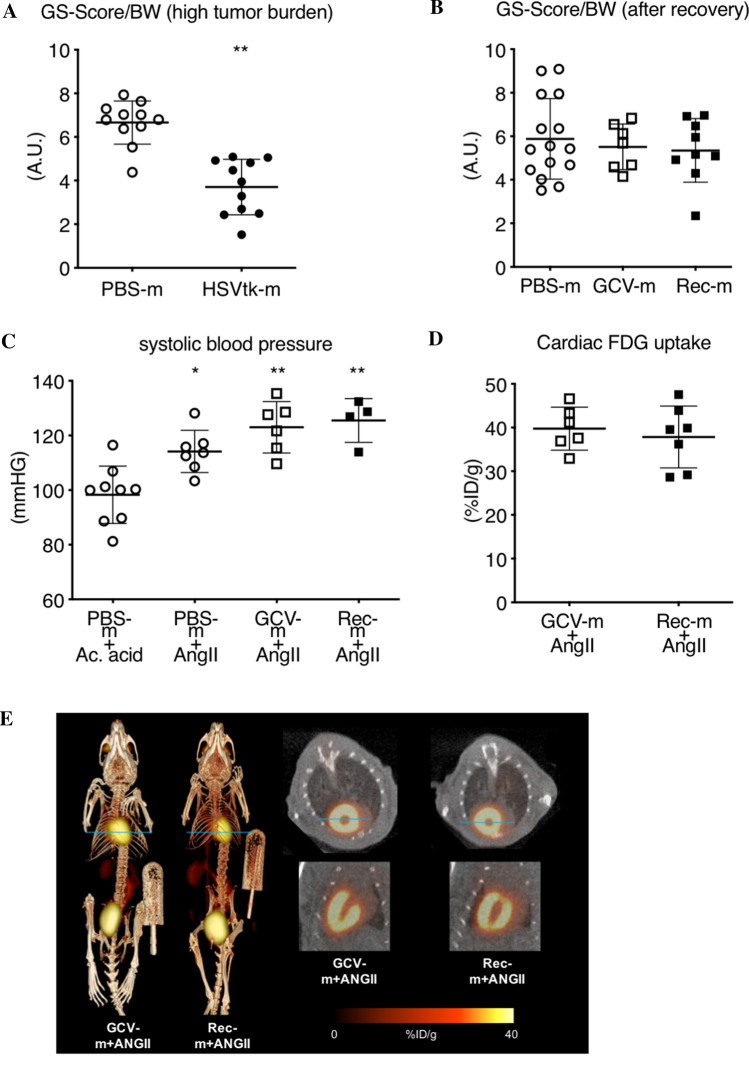
Physiological and pathophysiological stress tests after recovery from cancer. **A** Grip strength comparison between HSVtk-m (*n* = 11) at high tumor burden timepoint and accordingly timed PBS-m (*n* = 11). **B** Grip strength comparison between PBS-m (*n* = 15), GCV-m (*n* = 7) and Rec-m (*n* = 9). **C** Systolic blood pressure at chronic acetic acid (*n* = 9) and AngII stimulation in PBS-m (*n* = 7), GCV-m (*n* = 6) and Rec-m (*n* = 4). **D** Cardiac ^18^F-FDG quantitative uptake (percentage injected dose per gram tissue [%ID/g]) in GCV-m (*n* = 6) and Rec-m (*n* = 7) after AngII stimulation for 14 days. **E** Representative whole body and short and long-axis myocardial ^18^F-FDG PET/CT images from in GCV-m and Rec-m after AngII stimulation for 14 days. Data are depicted as mean ± SD, *P < 0.05, **P < 0.01 vs. respective PBS-m control group using either 1-way ANOVA followed by Bonferroni post hoc tests or two-tailed Student’s *t* test as required

Impaired physical abilities and muscle weakness are common in patients with advanced tumor disease [7]. Diminished muscle strength was also observed in HSVtk-m at advanced tumor stage (Fig. 3A). Rec-m displayed complete recovery of muscle strength compared to PBS-m and GCV-m controls demonstrating that a former cancer disease and muscle atrophy mediates no long-lasting negative effects on the physical resilience/strain (Fig. 3B).

### Response to pathophysiological stress in mice recovered from advanced tumor disease

High blood pressure is a frequent comorbidity in long-term survivors of cancer disease [3]. To investigate the cardiac exercise tolerance in a pathophysiological stress situation after a former cancer disease, we explored the tolerance to increased blood pressure in Rec-m by continuous AngII infusion (via osmotic minipumps). As shown in Fig. 3C AngII increased blood pressure in Rec-m and GCV-m controls to a similar degree when compared to healthy vehicle (acetic acid)-treated controls. Furthermore, AngII-induced cardiac hypertrophy (HW/BW ratio) was increased and cardiac function was reduced to a similar degree in Rec-m + AngII and GCV-m + AngII compared to GCV-m controls (Tab. 3). Also, AngII-mediated cardiac ^18^F-FDG uptake was comparable in Rec-m and GCV-m controls (Fig. 3D, E).

**Table 3:** Pathophysiological stress tolerance in recovered mice

	GCV-m (*n* = 7)	GCV-m + AngII (*n* = 7)	Rec-m + AngII (*n* = 7)
HW (mg)	116.1 ± 10.2	122.7 ± 10.7	120.9 ± 16.6
BW (g)	32.2 ± 3.1	24.4 ± 1.1**	23.0 ± 1.6**
HW/BW (mg/g)	3.6 ± 0.2	5.1 ± 0.3**	5.3 ± 0.4**
FS (%)	38.3 ± 1.3	27.1 ± 7.5**	25.2 ± 6.1**
HR (bpm)	599 ± 23	591 ± 22	568 ± 36
LVEDD (mm)	7.6 ± 0.4	7.1 ± 0.4	7.1 ± 0.3
LVESD (mm)	6.1 ± 0.4	5.8 ± 0.4	5.8 ± 0.4
CO (ml/min)	20 ± 2.3	14 ± 2.2**	13 ± 1.8**
ESV (μl)	33.5 ± 3.9	24.1 ± 3.1**	22.7 ± 2.3**
EVd (μl)	60.2 ± 8.4	43.6 ± 6.3**	44.1 ± 6.3**
EVs (μl)	26.6 ± 5.7	19.6 ± 4.2	21.4 ± 6.9

Data are depicted as mean ± SD, **P < 0.01 vs. GCV-m, using one-way ANOVA followed by Bonferroni post hoc tests

*HW/BW* heart weight (HW), body weight (BW), *FS* fractional shortening, *HR* heart rate, *LVEDD* left ventricular end-diastolic diameter *LVESD* left ventricular end-systolic diameter, *CO* cardiac output, *ESV* endocardial stroke volume, *EVd* endocardial volume diastolic, *EVs* endocardial volume systolic, from GCV-treated tumor-free control mice with (GCV-m + AngII) or without (GCV-m) angiotensin II (Ang II) treatment and recovered mice with AngII (Rec-m + AngII)

### Alterations in the cardiac gene expression profile at advanced cancer stage and after tumor elimination

**Fig. 4:**
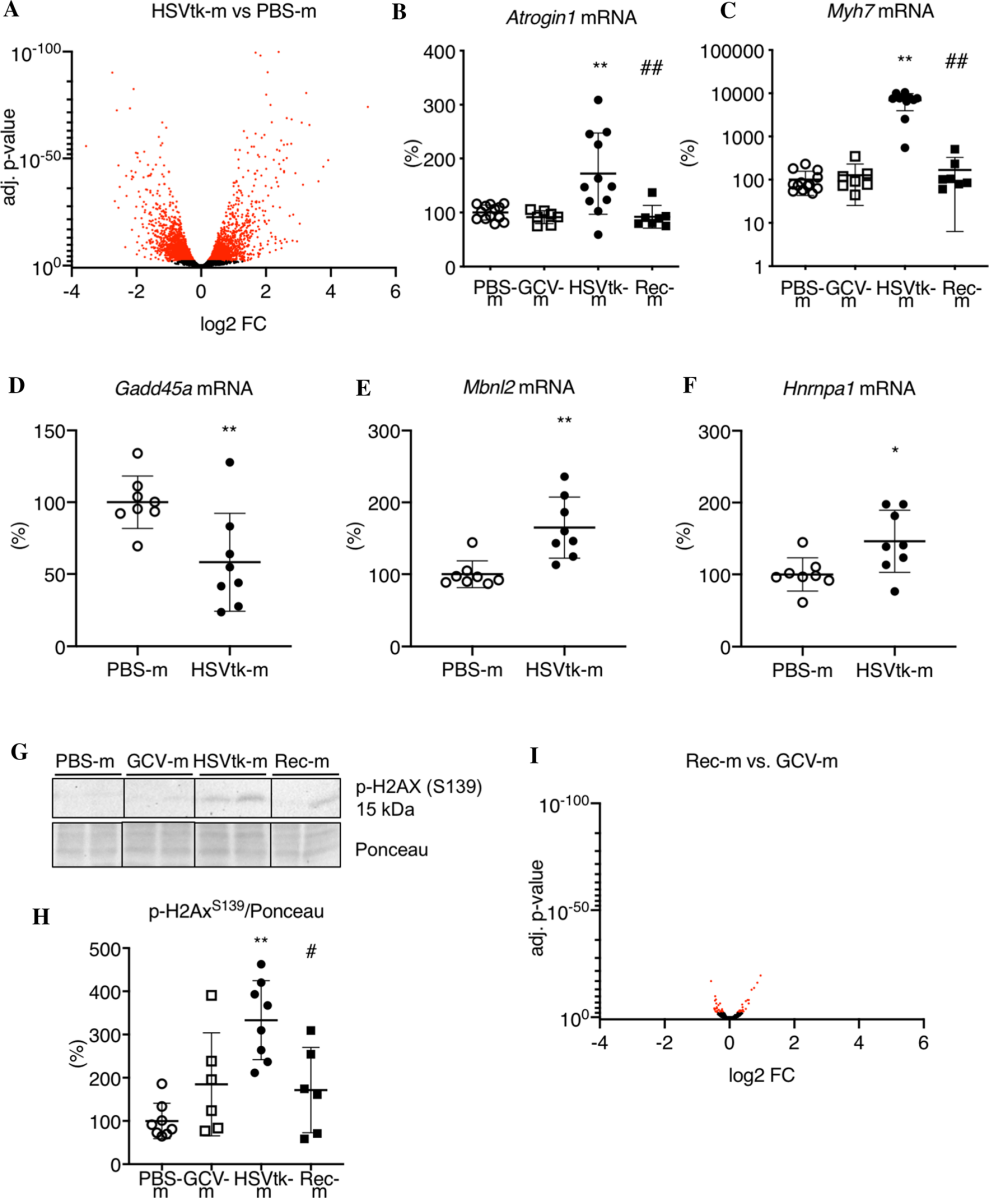
Changes in LV RNA expression at high tumor burden and after recovery from cancer. **A** Volcano plot of log2 fold change vs. adj. *P* value data from RNA-Seq. of LV tissue of PBS-m compared to HSVtk-m with adj. *P* value ≤ 0.01 highlighted in red, (filter of a base mean read count of ≥ 100, an adjusted *P* value ≤ 0.01 vs. healthy PBS-m; *n* = 2–4 individuals were pooled resulting in 3 pool samples per group). **B**, **C** Quantitative mRNA levels (qRT-PCR) of Atrogin1 and Myh7 in LV tissue from PBS-m (*n* = 13), GCV-m (*n* = 7), HSVtk-m (*n* = 11) and Rec-m (*n* = 7) normalized to 18S. **D**–**F** Quantitative mRNA levels (qRT-PCR) of *Gadd45a*, Mbnl2 and *Hnrnpa1* in LV tissue from PBS-m (*n* = 8) and HSVtk-m (*n* = 8) normalized to 18S. **G**, **H** Representative western blot images of P-H2AX from LV tissue of PBS-m (*n* = 6) vs. (*n* = 6) and corresponding quantification normalized on Ponceau staining. **I** Volcano plot of log2 fold change vs. adj. *P* value data from RNA-Seq. of LV tissue of GCV-m compared to Rec-m with adj. *P* value ≤ 0.01 highlighted in red (filter of a base mean read count of ≥ 100, an adjusted *P* value ≤ 0.01 vs. healthy GCV-m; *n* = 2–4 individuals were pooled resulting in 3 pool samples per group). Data are depicted as mean ± SD, *P < 0.05, **P < 0.01 vs. respective non-tumor group and ^##^*P* < 0.01 vs. respective non-GCV group using two-way ANOVA followed by Bonferroni post hoc tests or two-tailed-Student’s t test as required

The gene expression pattern of LV tissue from HSVtk-m with advanced tumor disease compared to healthy PBS-m controls was assessed by RNA-Seq. and revealed significant alterations in 3724 transcripts (of total 38,087 measured transcripts) with 1797 up- and 1927 downregulated (Fig. 4A, Table 4). A subset of altered mRNAs (*Myh7*, *Atrogin1*, *Irs2*) were confirmed by qRT-PCR (Figs. 2E, 4B, C). Bioinformatic analyses (DAVID functional annotation tool) revealed that 87 pathways with 10 or more differently expressed genes were affected including metabolic pathways, PI3K-AKT-Signaling and pathways in cancer as the most prominent ones (Table 4, Suppl. Fig. 3). In addition, DNA-Damage induced *Gadd45a* mRNA was significantly reduced in LV tissue and *Mbnl2* and *Hnrnpa1* mRNA, generally associated with fetal splicing pattern, were significantly increased (Fig. 4 D–F), which was associated with increased protein expression of the DNA damage marker p-H2AX in HSVtk-m (Fig. 4G, H, Suppl. Fig. 4A, B). In contrast, Rec-m showed a similar LV protein expression of p-H2AX compared to GCV-m (Fig. 4G, H, Suppl. Fig. 4A, B). Next, we compared the cardiac gene expression profile between Rec-m and control GCV-m and observed that only 1.6% (58 vs. 3724 genes) of the transcripts were differentially expressed, and altered pathways from the advanced disease stage had all normalized (Table 4, Suppl. Fig. 3A, B).

**Table 4:** DAVID-based analyses of RNA-Seq. from left ventricular tissue of tumor-bearing and recovered mice

KEGG-pathway analysis with functional annotation tool (DAVID bioinformatics resources)	Gene count after filter criteria	Max. count of genes per pathway	Count of pathways with at least 10 diff. expr. genes
**HSVtk-m vs. PBS-m**	3724	351	87
Increased expression	1797	69	44
Reduced expression	1927	258	61
**Rec-m vs. GCV-m**	58	5	0
Increased expression	25	4	0
Reduced expression	33	0	0

RNA-Seq. data were pre-filtered with a filter of a base mean read count of ≥ 100 and an adjusted *P* value ≤ 0.01 vs. healthy PBS-m respectively GCV-m controls. Pre-filtered RNA-Seq. data gene list was analysed with the DAVID functional annotation tool based on KEGG pathways

### Impact of doxorubicin on survival and recovery in Rec-HSVtk-m

**Fig. 5:**
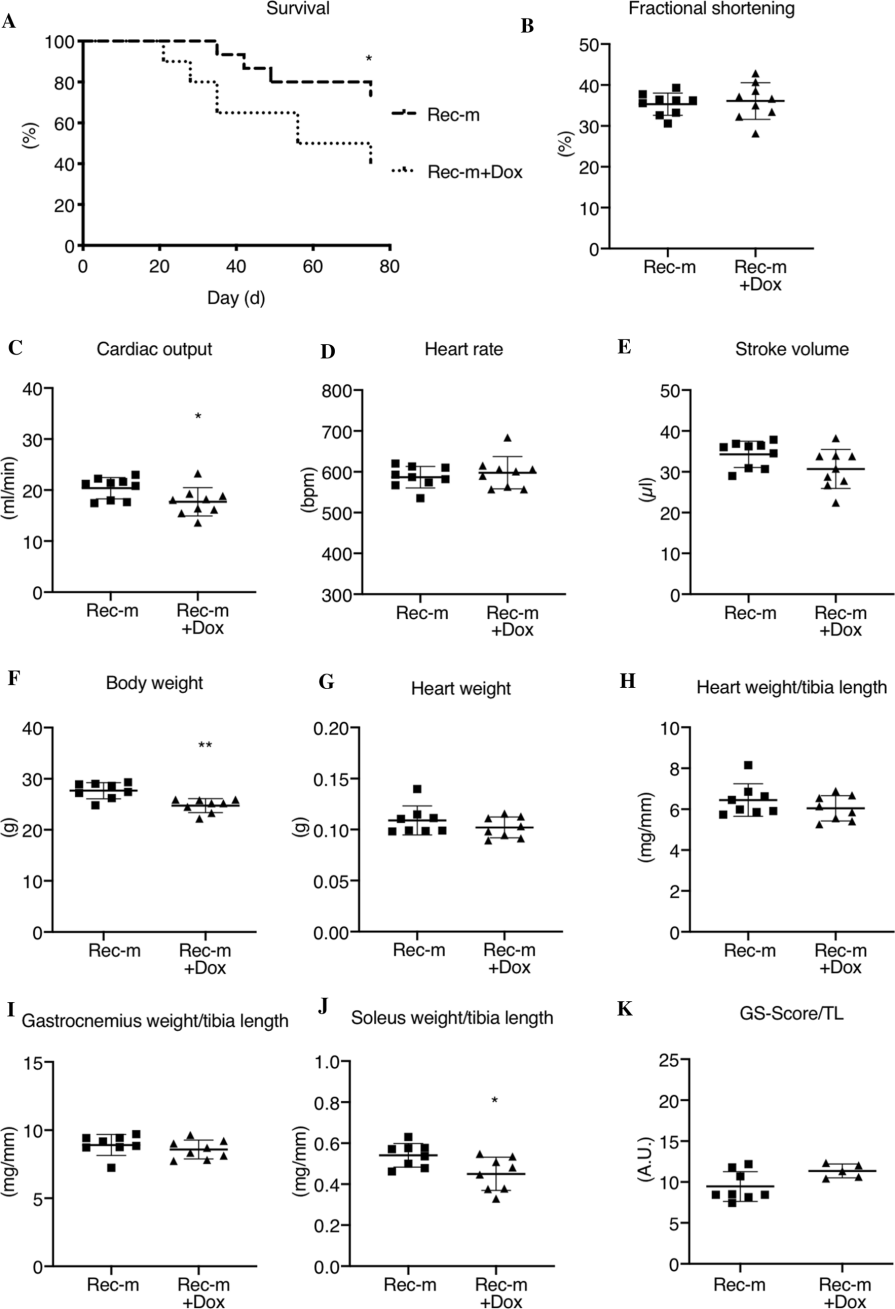
Cardiac phenotype after recovery from cancer and doxorubicin therapy. **A** Percent survival comparison of Rec-m (*n* = 15) and Rec-m + Dox (*n* = 20) from HSVtk-c injection until day of tissue harvesting. B-E) Fractional shortening, cardiac output, heart rate and stroke volume from echocardiographic analyses of Rec-m (*n* = 9) and Rec-m + Dox (*n* = 9). **F**–**J** Body weight and heart weight, gastrocnemius weight and soleus weight per tibia length from Rec-m (*n* = 8) and Rec-m + Dox (*n* = 8). **K** GS-Score per tibia length from Rec-m (*n* = 8) and Rec-m + Dox (*n* = 5). Data are depicted as mean ± SD, *P < 0.05, **P < 0.01 vs. Rec-m using Log-rank Mantel-Cox test, 2-tailed-Student’s *t* test or Mann–Whitney test as required

Anti-cancer treatment with the AC Dox is associated with acute and long-term cardiotoxic effects in cancer patients [45]. To mimic the Dox treatment of cancer disease in mice, HSVtk-m with advanced tumor disease were treated with Dox (3 doses of 4 mg/kg), which led to a decrease in the tumor signal in IVIS (Suppl. Fig. 5A). In addition, B16F10-HSVtk cells react sensitive to Dox treatment in vitro (Suppl. Fig. 5B). Since Dox alone did not completely eliminate tumors in HSVtk-m, an additional treatment with GCV was added until tumor signal was lost to allow analyses after recovery from cancer disease (Rec-m + Dox). Dox treatment followed by complete tumor elimination through GCV (Rec-m + Dox) was associated with an improved survival rate compared to untreated tumor bearing mice (HSVtk). In contrast, Dox treatment reduced survival of Rec-m + Dox significantly compared to Rec-m not treated with Dox (Fig. 5A).

In surviving and recovered mice, cardiac function was comparable between Rec-m with and without Dox treatment but CO was slightly reduced at a comparable heart rate and SV in the Dox-treated group (Fig. 5B–E). Furthermore, Dox-treated Rec-m showed significantly lower BW and SW/TL compared to Rec-m while HW, HW/TL and GW/TL were not altered (Fig. 5 F-J). Overall grip strength was assessed and normalized to TL due to the BW difference but was not significantly altered between Rec-m with and without Dox treatment (Fig. 5K). In addition, GLUT4 and GLUT1 mRNA expression was unchanged in Rec-m with and without Dox treatment (Suppl. Fig. 5C, D).

### Doxorubicin persistently altered the cardiac gene expression program in mice recovered from tumor disease

**Fig. 6:**
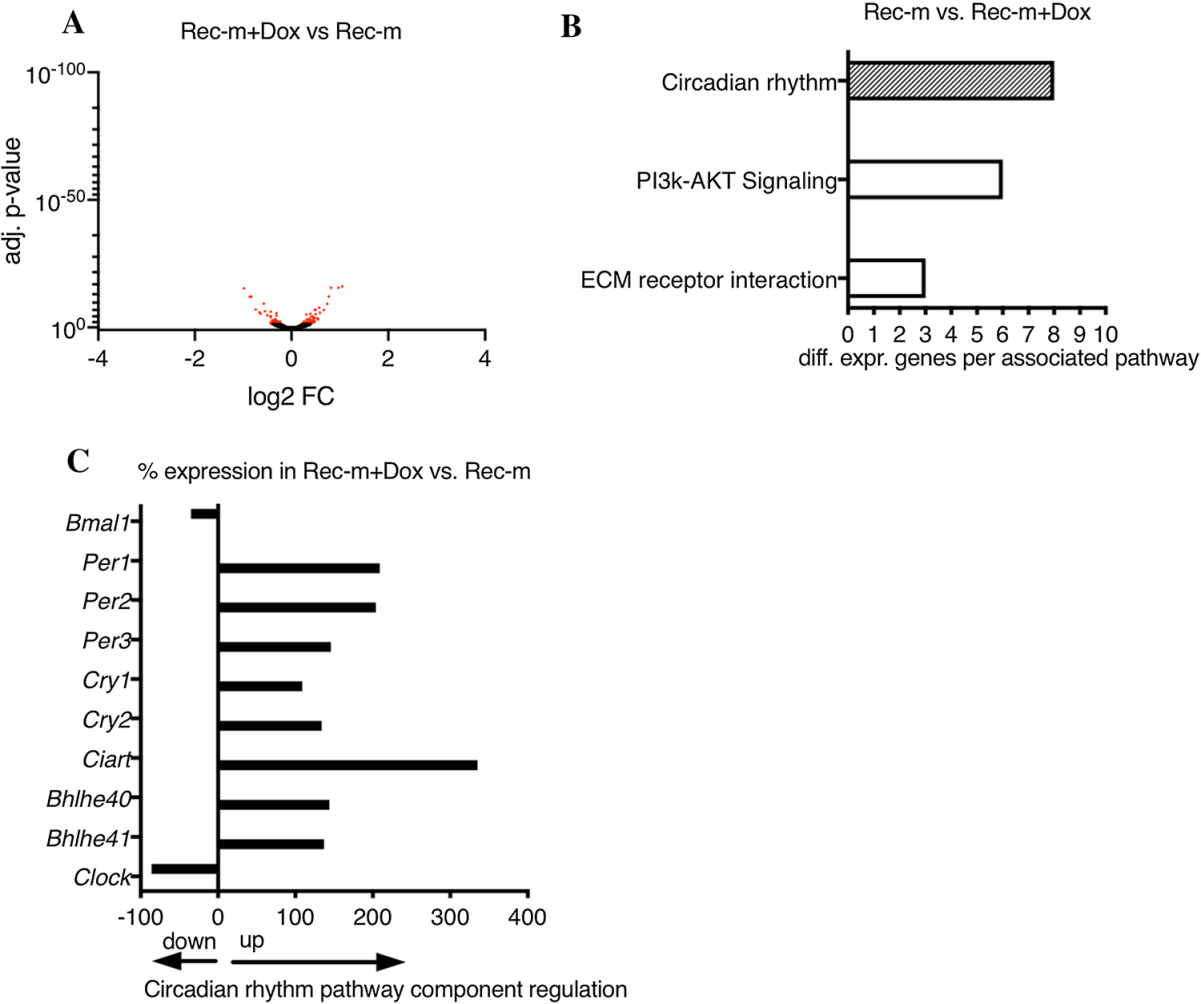
Doxorubicin-induced changes in cardiac circadian rhythm. **A** Volcano plot of log2 fold change vs. adj. *P* value data from RNA-Seq. of LV tissue of Rec-m compared to Rec-m + Dox with adj. *P* value ≤ 0.05 highlighted in red. (filter of a base mean read count of ≥ 100, an adjusted *P* value ≤ 0.05 vs. healthy Rec-m; *n* = 2–4 individuals were pooled resulting in 3 pool samples per group) **B** DAVID pathway analyses of significantly different expressed genes in RNA-Seq. data comparison of Rec-m and Rec-m + Dox. **C** Regulation percentage of circadian rhythm pathway components in Rec-m + Dox in comparison to Rec-m

RNA-Seq. on recovered Dox treated Rec-m LVs revealed 123 genes changed between Rec-m with and without Dox treatment with 64 up- and 59 downregulated (Fig. 6A). DAVID-based pathway analysis of significantly different expressed genes discovered the circadian rhythm pathway as the most altered between Rec-m with and without Dox treatment, wherein central components including *Bmal1* and *Clock* were reduced and *Period1-3*, *Cryptochrome1/2 (Cry1/2)*, circadian associated repressor of transcription (*Ciart*) and basic helix–loop–helix family members 40/41 (*Bhlhe40/41*) were increased in the heart (Fig. 6B, C). The expression pattern of *Clock, Bmal1, Cyr1* and *Cry2* induced by Dox in skeletal muscle (SKM) from the same animals was not altered (Suppl. Fig. 6A–F) but *Per2* and *Ciart* were significantly increased as also observed in the heart tissue (Suppl. Fig. 6B, E).

### Impairment in the expression of circadian rhythm genes leads to elevated induction of cardiomyocyte apoptosis under doxorubicin treatment

**Fig. 7:**
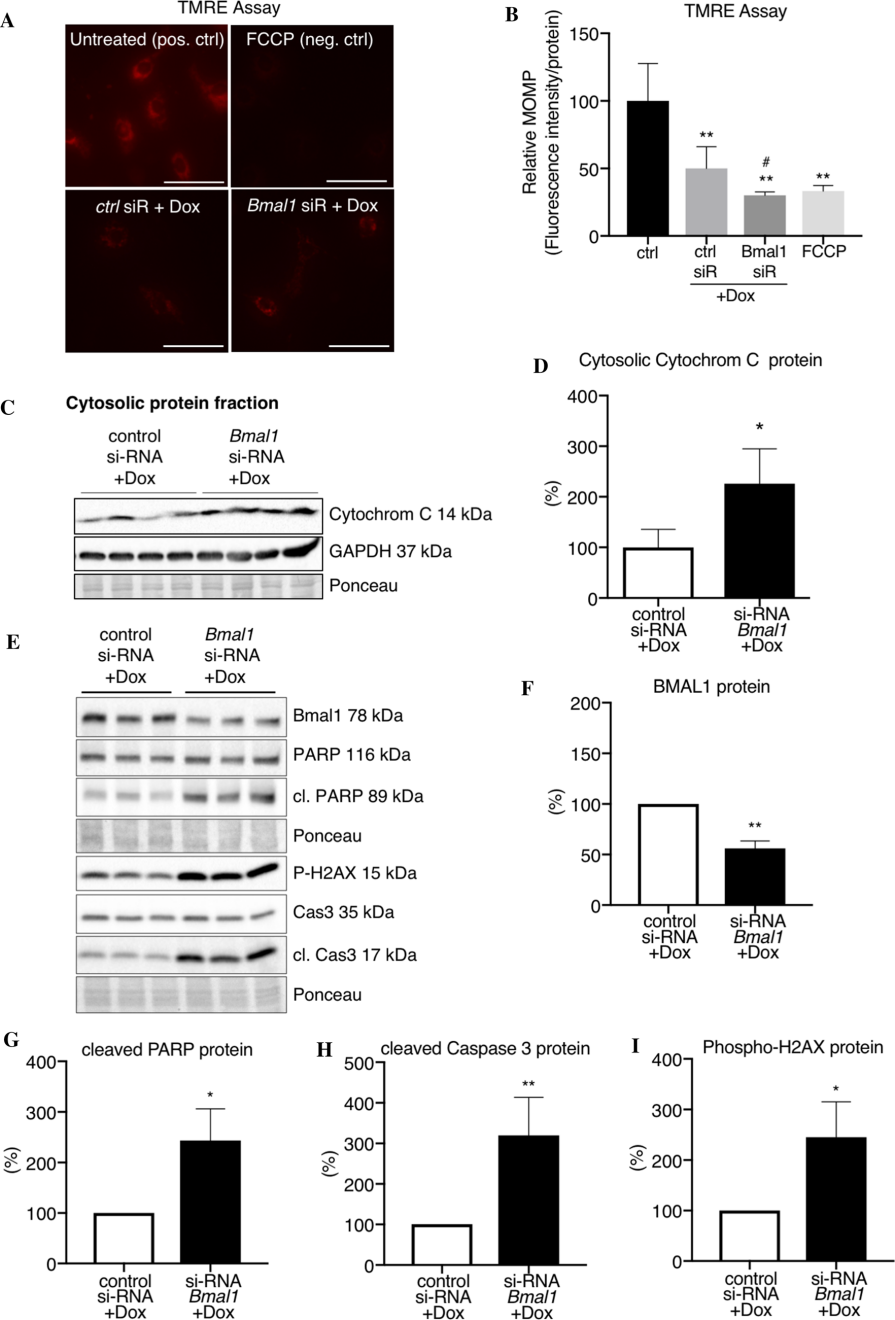
Doxorubicin-induced increased apoptosis in NRCM with siRNA-mediated knockdown of BMAL1. **A** Representative images of TMRE in NRCM treated with Dox for 24 h after pre-treatment with *Bmal1*-si-RNA or scrambled siR control for 48 h, scale bar indicates 50 μm. FCCP (carbonyl cyanide 4-(trifluoromethoxy) phenylhydrazone) served as negative control and untreated NRCM as positive control. **B** Quantification of relative TMRE-mitochondrial membrane potential (MOMP) in living NRCM after treatment with *Bmal1*-si-RNA or scrambled siR-control for 48 h followed by 24 h Dox normalized to total protein concentration, *n* = 4–5 per group. FCCP served as negative control and untreated NRCM as positive control. **C**, **D** Representative western blot images of cytochrome C and GAPDH protein from the cytosolic protein fraction of NRCM after treatment with *Bmal1*-si-RNA or scrambled siR-control for 48 h followed by 24 h Dox and corresponding quantification normalized on Ponceau staining from *n* = 4 pooled samples per group (each pool consists of 2 individual samples). **E**–**I** Representative western blot images of BMAL1, PARP, cleaved PARP, p-H2AX, Caspase 3 and cleaved Caspase 3 protein from NRCM transfected with *Bmal1*-si-RNA or scrambled siR-control for 48 h followed by 24 h Dox and corresponding quantification normalized on Ponceau staining from *n* = 4–6 independent experiments (each set contains of 3–4 replicates that were summarized and the control condition of each experiment was set to 100%). Data are depicted as mean ± SD, *P < 0.05, **P < 0.01 vs. siRNA control, using one-sample-*t*-test

Since it is known that the circadian rhythm impacts on the cardiac sensitivity to Dox treatment, and given its central role in the circadian rhythm regulation, *Bmal1* was targeted in an si-RNA-based approach to assess apoptosis induction due to a subsequent Dox stimulation in neonatal rat cardiomyocytes (NRCM) (Fig. 7A–I, Suppl. Figs. 7A, B, 8A–J). NRCM with si-RNA-mediated BMAL1 downregulation revealed a significantly reduced mitochondrial membrane potential measured by tetramethylrhodamine, ethyl ester (TMRE) assay, which was associated with an increased release of cytochrome C into the cytosol after Dox treatment compared to control-siR-treated NRCM (Fig. 7A–D, Suppl. Fig. 8A–C). The occurrence of apoptotic NRCM was quantified by in situ cell death detection/TUNEL assay, which revealed an increased ratio of TUNEL^+^/DAPI^+^ NRCMs in BMAL1 siR + Dox compared to control-siR-treated NRCM (Suppl. Fig. 7A, B). In addition, NRCM with si-RNA mediated BMAL1 downregulation showed increased Caspase 3 and PARP cleaving and an increased expression of the DNA damage marker p-H2AX after Dox treatment compared to control-siR-treated NRCM (Fig. 7E-I, Suppl. Fig. 8D-J).

## Discussion

In the present study, we developed a reversible murine cancer model allowing for live in vivo visualization of tumor growth and enabling cancer-cell-specific anthracycline-free elimination of cancer cells by GCV treatment. Using this model, we observed that almost all cancer-induced phenotypic, metabolic, molecular and functional alterations of the heart were reversible after anthracycline-free tumor elimination and recovery. Moreover, physical strength and tolerance to AngII-induced high blood pressure was normal in recovered tumor mice demonstrating that additional stress factors (as part of the second hit hypothesis) are not inducing impaired cardiac performance. In turn, administering Dox prior GCV-induced complete tumor elimination, resulted in high mortality in tumor-bearing mice. However, in surviving mice, cardiac phenotype and function were largely normalized though at the molecular level permanent alterations in the cardiac gene expression pattern with a prominent change in the circadian rhythm pathway were observed. Thus, the heart is able to recover and regenerate from cancer-induced cardiac damage almost completely, while Dox promotes high mortality in the acute phase and long-term subclinical changes in the heart.

To analyse the capacity for recovery of the heart after advanced cancer disease, we developed a mouse model for melanoma in which effective tumor-cell killing could be monitored by signal loss in IVIS. Subsequently, recovery of cardiac function, dimensions and heart weight was measured. We observed that cardiac morphology and function returned to age-matched tumor-free controls with normalization of cardiomyocyte dimensions and no traces of cardiac tissue damage such as inflammation or fibrosis, a feature that was also confirmed by RNA-Seq. analysis on the molecular level. Along with the increase in cardiac and skeletal muscle weights, Rec-m showed comparable grip strength to tumor-free controls indicating not only cardiac improvement but also a high degree of recovery of the overall condition. This is also supported by the similar reactions to AngII stimulation inducing mild functional decrease in association with increased heart weight to body weight ratio and comparable ^18^F-FDG uptake independent of former systemic advanced melanoma burden. Beside complete normalization of heart function, and largely normalized morphological and molecular phenotype of the heart, the physical stress and the induced pressure overload support functional, morphological analyses that no subclinical impairment of the heart remained.

Previous reports indicate also substantial impairment in systemic and cardiac insulin signalling during advanced cancer disease associated with reduced protein kinase B (AKT) activation [34, 43]. AKT acts as a nodal point affecting substrate uptake as well as balancing anabolic and catabolic cardiac pathways. Rec-m displayed increased levels of circulating insulin and improved activation of AKT (phosphorylation at Ser^473^) albeit not reaching levels of PBS-m and GCV-m. Due to the initial spread of melanoma cells after intraperitoneal injection, it is likely that irreversible structural damage of pancreatic tissue has occurred, which may explain a persistent reduction of plasma insulin levels and subsequent lower AKT activation in the heart. Despite lower systemic insulin and AKT activation cardiac *Irs2* and CD36 were completely normalized, indicative of sufficient insulin signalling and metabolic recovery of the heart. As a limitation of this study, it has to be stated that cardiac ^18^F-FDG uptake was not analysed in tumor-bearing mice as we had previously shown that melanoma tumor burden reduces cardiac ^18^F-FDG uptake [43]. Metabolic recovery was further supported by the normalization of blood hexoses reduction in Rec-m. Solid tumors are known to degrade plasma abundant lysophosphatidylcholines (lysoPC) to utilize free fatty acids for membrane integration [38]. Similarly, sphingomyelinsynthase activity is often increased in tumors to enhance synthesis of sphingolipids with pro-survival functions [23]. Moreover, elevated sarcosine levels are associated with Alzheimer, dementia, prostate cancer, colorectal cancer or stomach cancer [36]. Here, the observed alterations in lysoPCs, sphingomyelins (SM), and sarcosine level at advanced cancer disease stage and their normalization after recovery in Rec-m confirm the advanced cancer stage and the complete recovery thereafter. Interestingly, carnitine levels were reported to be reduced in childhood cancer survivors with cardiac dysfunction that received chemotherapy with a mean of 12 years prior to the study [2]. In our model, we additionally found carnitine reduction in plasma samples during advanced cancer stage but normalization after recovery, indicating that persistent metabolic alterations might be related to the combined or exclusive effects of anthracyclines used during oncologic treatment.

RNA-Seq. and qRT-PCR analyses also revealed that most cancer-induced expression changes were normalized after the recovery phase. Among normalized genes were *Hnrnpa1* and *Mbnl2* mRNA expression, whose expression was significantly increased in hearts during advanced tumor burden. Both are involved in fetal splicing activities, and *Hnrnpa1* upregulation is known to be involved in an imbalance of RNA-processing factors causing myotonic dystrophy type 1 (DM1) [26]. Changes in transcript expression of DNA repair and damage genes were associated with an increased expression of phosphorylated-H2AX, a marker for DNA damage, at advanced cancer stages.

To analyse the potential specific long-lasting effect of anthracycline-based chemotherapy on the cancer-damaged heart (and organism), we confirmed B16F10 sensitivity to Dox and treated HSVtk-m with a moderate dose, which markedly reduced tumor burden but did not eliminate all cancer cells. Therefore, we continued the Dox treatment with GCV until the tumor signal was gone. Dox treatment was associated with an increased mortality in the acute phase, which was not observed in HSVtk-m treated with GCV alone. Moreover, surviving Rec-m + Dox mice displayed lasting BW and SW loss and a mild reduction in cardiac output at follow-up, although systolic function was normal and the physiological stress test (weight lifting) was comparable. Unfortunately, the effect of Dox on long-term recovery and pathophysiological stress tolerance, i.e. high blood pressure could not be tested in our mouse model due to ethical concerns of too many severe interventions in the same experimental animals. However, in the literature, there is evidence that Dox treatment and high blood pressure promote cardiovascular disease and heart failure in cancer survivors [4, 24]. In addition, Rec-m + Dox mice revealed substantial changes of the circadian rhythm pathway in LV tissue. Importantly, the circadian clock-controlled gene expression is relevant for maximizing anti-cancer efficacy of the pharmacological treatment as well as for minimizing sensitivity to treatment related cardiotoxicity [1]. Among the circadian genes permanently reduced by Dox in LV tissue was *Bmal1*, an interesting feature since *Bmal1*-KO mice develop age-dependent dilated cardiomyopathy [25]. Here we could show that reduction of BMAL1 expression in cardiomyocytes significantly increased their sensitivity to Dox-induced apoptosis. Furthermore, *Per2,* which was increased by Dox and also increased by advanced tumor disease, is associated with endothelial dysfunction and AKT-dependent senescence [37]. Similar to our observations, juvenile treatment with Dox was reported to change circadian clock-controlled gene expression in the heart, and these changes persisted into adulthood [12]. Also, for the human system, the relevance of circadian clock-controlled gene expression regarding anthracycline cardiotoxicity could be demonstrated in embryonic stem cell-derived cardiomyocytes, which showed an oscillating circadian pattern in the strength of the apoptotic response after Dox treatment [11], confirming our notion that combined effects of the underlying cancer disease and the oncological treatment should be considered when analyzing cancer-related cardiotoxicity [34]. A long-term disturbance in cardiac circadian rhythm with associated reduced stress tolerance might, therefore, also play a role in late-onset cardiotoxicity if considered as one of multiple ‘hits’ next to certain forms of genetic predisposition or cardiac stress events like pregnancy, [32, 33], high blood pressure or additional cardiotoxic treatment in relapse or recurrence of cancer disease.

In conclusion, cancer-induced cardiac alterations and damage are severe but widely reversible in the analysed tumor model without evidence for a contribution to long-term cardiac damage. However, the addition of cardiotoxic treatment with Dox impairs recovery from cancer-induced cardiac alterations by induction of long-lasting changes in the cardiac gene expression program even if cardiac systolic function seems normalized and thereby may render the heart more sensitive to additional stress during lifetime, which, at least in part, could contribute to late cardiac toxicity.

## Supplementary Information

Below is the link to the electronic supplementary material.

Supplementary file1 Suppl. Fig. 1: Whole western blots of Figure 2. Whole gel images of representative western blots shown in Fig. 2C of A) phospho-(Ser473) and B) total AKT from LV tissue of PBS-m, GCV-m, HSVtk-m and Rec-m and corresponding quantification normalized on C) Ponceau staining, and in Fig. 2F of D) CD36 from LV tissue of PBS-m, GCV-m, HSVtk-m and Rec-m and corresponding quantification normalized on E) Ponceau staining. (TIFF 10263 KB)

Supplementary file2 Suppl. Fig. 2: Cardiac GLUT4 and GLUT1 mRNA expression after anthracycline-free tumor elimination and recovery from cancer. A, B) Quantitative transcript levels determined by RNA-Seq. of A) GLUT4 and B) GLUT1 in LV tissue from n=2-4 individuals were pooled resulting in 3 pool samples per group (for: PBS-m, GCV-m, HSVtk-m and Rec-m). Data are depicted as mean ± SD, *P < 0.05, **P < 0.01 vs. respective non-tumor group and #P < 0.05, ##P < 0.01 vs. respective non-GCV group using 2-way ANOVA followed by Bonferroni posthoc tests. (TIFF 10263 KB)

Supplementary file3 Suppl. Fig. 3: DAVID pathway analysis of cardiac tissue in tumor-bearing and cured mice. Exemplary pathway overview on diff. expressed genes per pathway after DAVID based analyses of RNA-Seq. data of LV tissue of A) PBS-m compared to HSVtk-m and B) GCV-m compared to Rec-m (filter of a base mean read count of ≥ 100, an adjusted p-value ≤ 0.01 vs. healthy PBS-m), n=2-4 individuals were pooled resulting in 3 pool samples per group (for: PBS-m, GCV-m, HSVtk-m and Rec-m). (TIFF 10263 KB)

Supplementary file4 Suppl. Fig. 4: Whole western blots of Figure 4. Whole gel images of representative western blots shown in Fig. 4H of A) phospho-H2AX (S139) from LV tissue of PBS-m and HSVtk-m and corresponding quantification normalized on B) Ponceau staining. (TIFF 10263 KB)

Supplementary file5 Suppl. Fig. 5: Dox sensitivity of B16F10-HSVtk cells. A) Exemplary images of Dox treatment (after 2 doses at day 12 after tumor cell injection) monitored by IVIS. B) Representative images of Dox treated B16F10-HSVtk cells basal, 48h and 72h after Dox treatment, scale bar indicates 50 μm. Quantitative transcript levels determined by RNA-Seq. of C) GLUT4 and D) GLUT1 in LV tissue from n=2-4 individuals were pooled resulting in 3 pool samples per group (for: Rec-m, Rec-m+Dox). (TIFF 10263 KB)

Supplementary file6 Suppl. Fig. 6: Changes in skeletal muscle (SKM) RNA expression after recovery from cancer with and without Dox treatment. A-F) Quantitative mRNA levels (qRT-PCR) of A) Bmal1, B) Per2, C) Cry1, D) Cry2, E) Ciart and F) CLOCK in SKM tissue from Rec-m (n=9) and Rec-m+Dox (n=7) normalized to 18S. Data are depicted as mean ± SD, *P < 0.05, **P < 0.01 vs. respective Rec-m group, using 2-tailed-Student’s t-test. (TIFF 10263 KB)

Supplementary file7 Suppl. Fig. 7: Doxorubicin-induced increased apoptosis in NRCM with siRNA-mediated knockdown of BMAL1. A) Representative images of TUNEL+ (green), DAPI+ (blue) and merged TUNEL+/DAPI+ from neonatal rat cardiomyocytes treated with Dox for 24 hours after pre-treatment with Bmal1-siRNA or scrambled siR-control for 48 hours, scale bar indicates 50 μm. B) Bar graph depicts the ratio of TUNEL+ to DAPI+ NRCM treated with Dox for 24 hours after pre-treatment with Bmal1-si-RNA or scrambled siR-control for 48 hours from n=3 experiments. Data are depicted as mean ± SD, *P < 0.05 vs. scrambled siR-control using 2-tailed-Student’s t-test. (TIFF 10263 KB)

Supplementary file8 Suppl. Fig. 8: Whole western blots of Figure 7. Whole gel images of representative western blots shown in Fig. 7C of A) cytochrome C, B) GAPDH from the cytosolic protein fraction of NRCM after treatment with Bmal1-si-RNA or scrambled siR-control for 48 hours followed by 24 hours Dox and corresponding quantification normalized on C) Ponceau staining, and in Fig. 7E of D) BMAL1, E) PARP, F) Ponceau, G) p-H2AX, H, I) caspase 3 and corresponding J) Ponceau staining from NRCM transfected with Bmal1-si-RNA or scrambled siR-control for 48 hours followed by 24 hours Dox. (TIFF 10263 KB)

Supplementary file9 (TIFF 10263 KB)
