# Supplementary figures and images for: Anthracycline-free tumor elimination in mice leads to functional and molecular cardiac recovery from cancer-induced alterations in contrast to long-lasting doxorubicin treatment effects
Source: Basic Res Cardiol. 2021 Oct 20;116(1):61. doi: 10.1007/s00395-021-00902-7 (PMC8528750; doi:10.1007/s00395-021-00902-7)

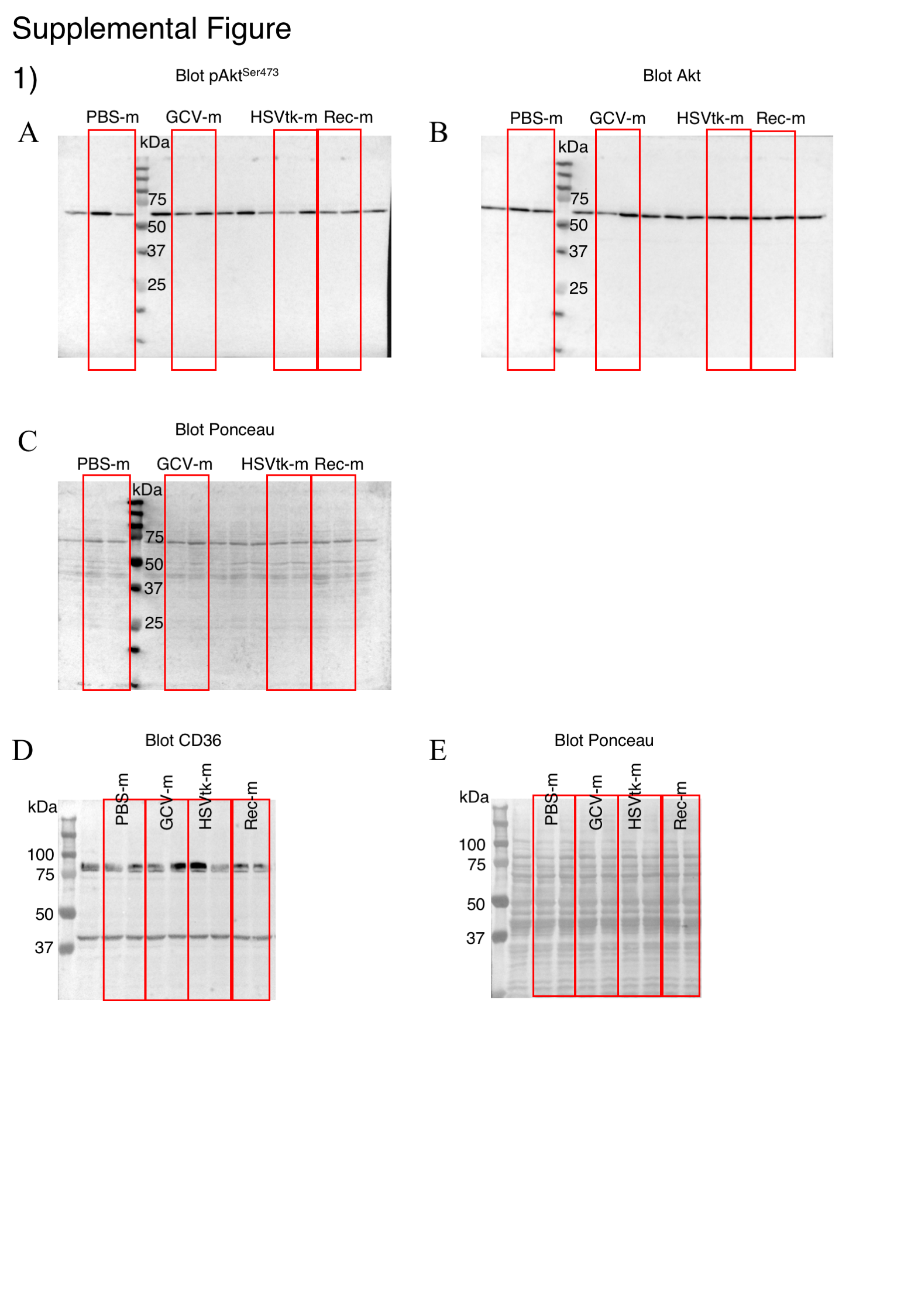

Supplement: Supplementary file 1 — Supplementary file1 Suppl. Fig. 1: Whole western blots of Figure 2. Whole gel images of representative western blots shown in Fig. 2C of A) phospho-(Ser473) and B) total AKT from LV tissue of PBS-m, GCV-m, HSVtk-m and Rec-m and corresponding quantification normalized on C) Ponceau staining, and in Fig. 2F of D) CD36 from LV tissue of PBS-m, GCV-m, HSVtk-m and Rec-m and corresponding quantification normalized on E) Ponceau staining. (TIFF 10263 KB) [file 395_2021_902_MOESM1_ESM.tiff]

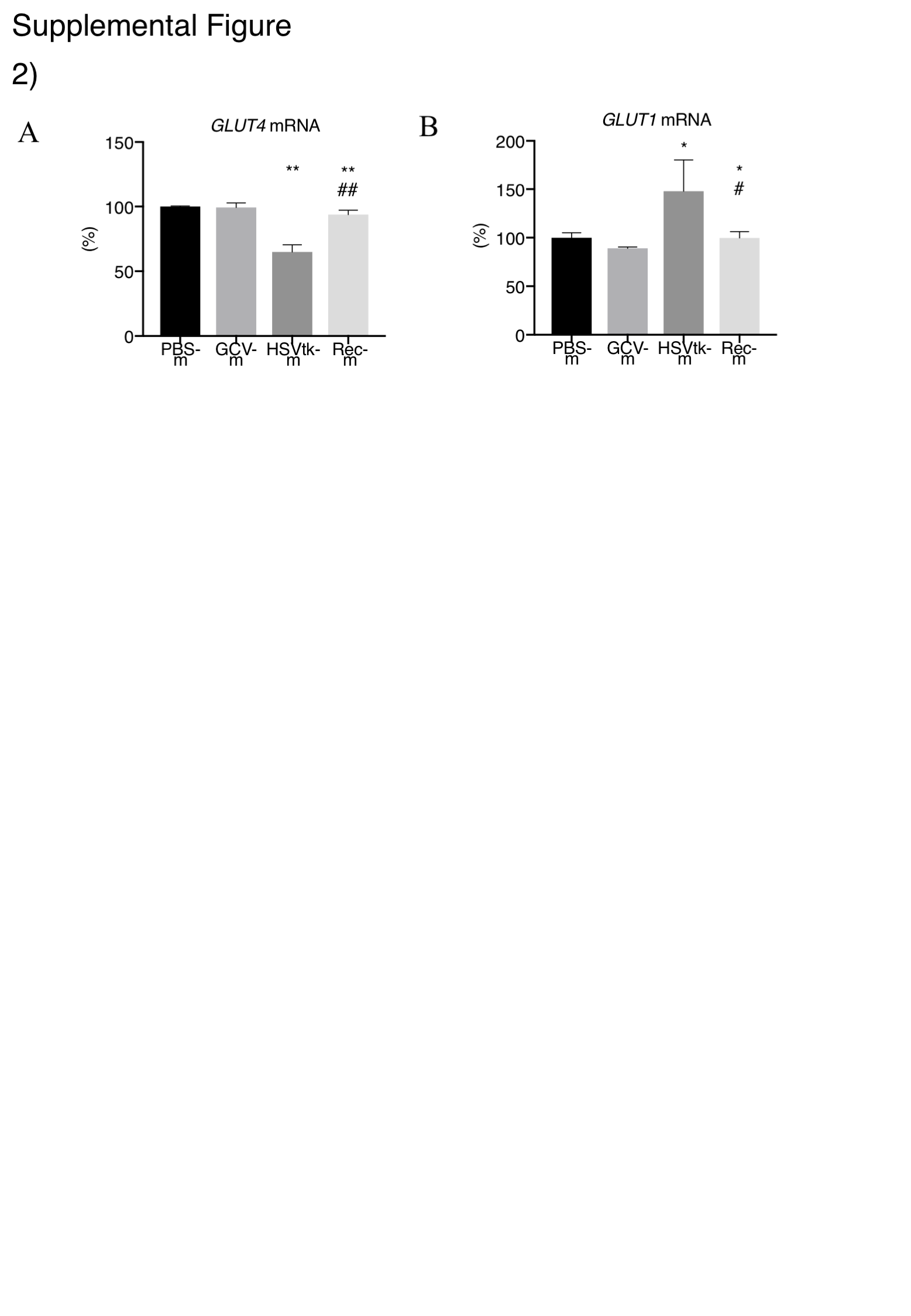

Supplement: Supplementary file 2 — Supplementary file2 Suppl. Fig. 2: Cardiac GLUT4 and GLUT1 mRNA expression after anthracycline-free tumor elimination and recovery from cancer. A, B) Quantitative transcript levels determined by RNA-Seq. of A) GLUT4 and B) GLUT1 in LV tissue from n=2-4 individuals were pooled resulting in 3 pool samples per group (for: PBS-m, GCV-m, HSVtk-m and Rec-m). Data are depicted as mean ± SD, *P < 0.05, **P < 0.01 vs. respective non-tumor group and #P < 0.05, ##P < 0.01 vs. respective non-GCV group using 2-way ANOVA followed by Bonferroni posthoc tests. (TIFF 10263 KB) [file 395_2021_902_MOESM2_ESM.tiff]

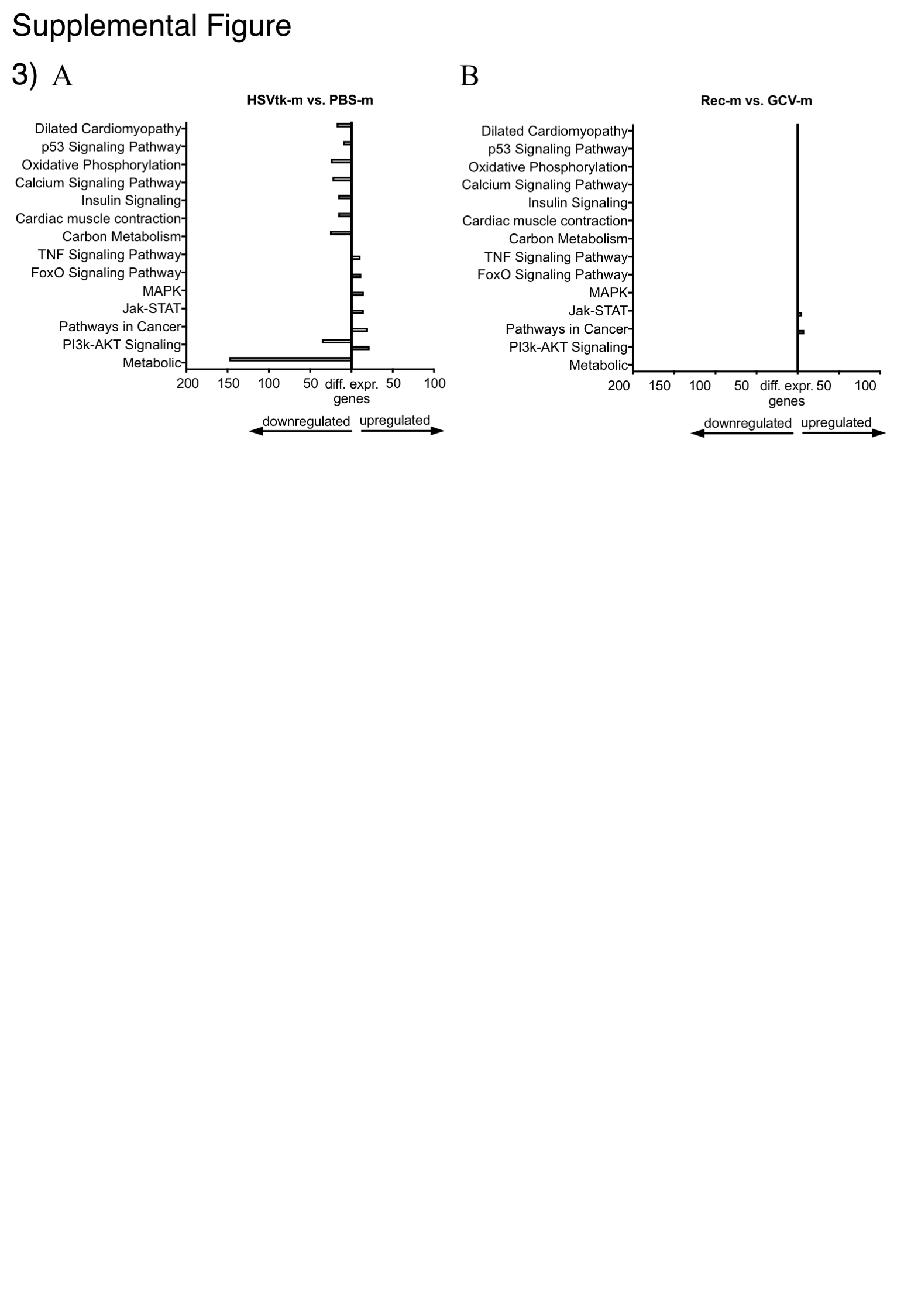

Supplement: Supplementary file 3 — Supplementary file3 Suppl. Fig. 3: DAVID pathway analysis of cardiac tissue in tumor-bearing and cured mice. Exemplary pathway overview on diff. expressed genes per pathway after DAVID based analyses of RNA-Seq. data of LV tissue of A) PBS-m compared to HSVtk-m and B) GCV-m compared to Rec-m (filter of a base mean read count of ≥ 100, an adjusted p-value ≤ 0.01 vs. healthy PBS-m), n=2-4 individuals were pooled resulting in 3 pool samples per group (for: PBS-m, GCV-m, HSVtk-m and Rec-m). (TIFF 10263 KB) [file 395_2021_902_MOESM3_ESM.tiff]

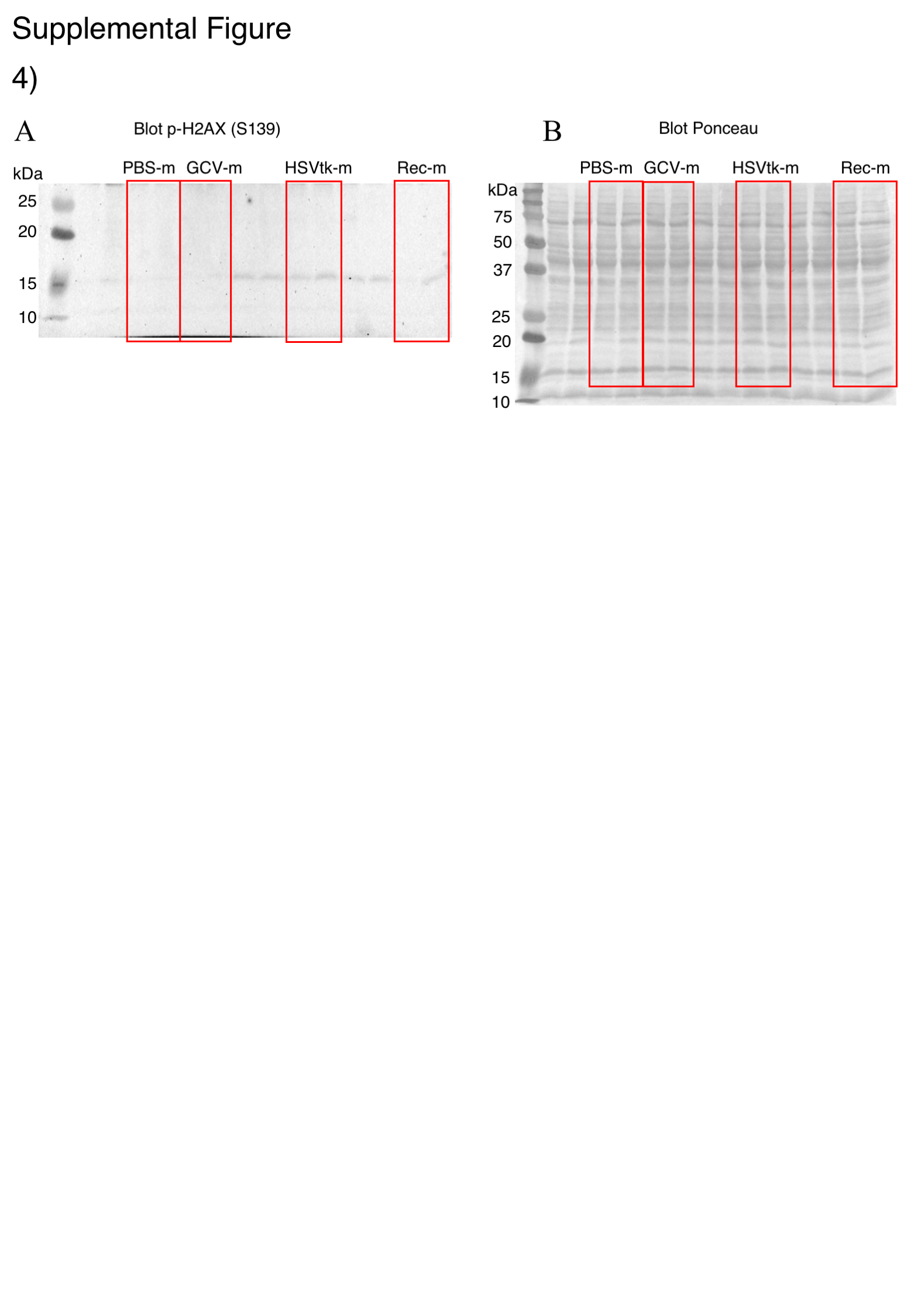

Supplement: Supplementary file 4 — Supplementary file4 Suppl. Fig. 4: Whole western blots of Figure 4. Whole gel images of representative western blots shown in Fig. 4H of A) phospho-H2AX (S139) from LV tissue of PBS-m and HSVtk-m and corresponding quantification normalized on B) Ponceau staining. (TIFF 10263 KB) [file 395_2021_902_MOESM4_ESM.tiff]

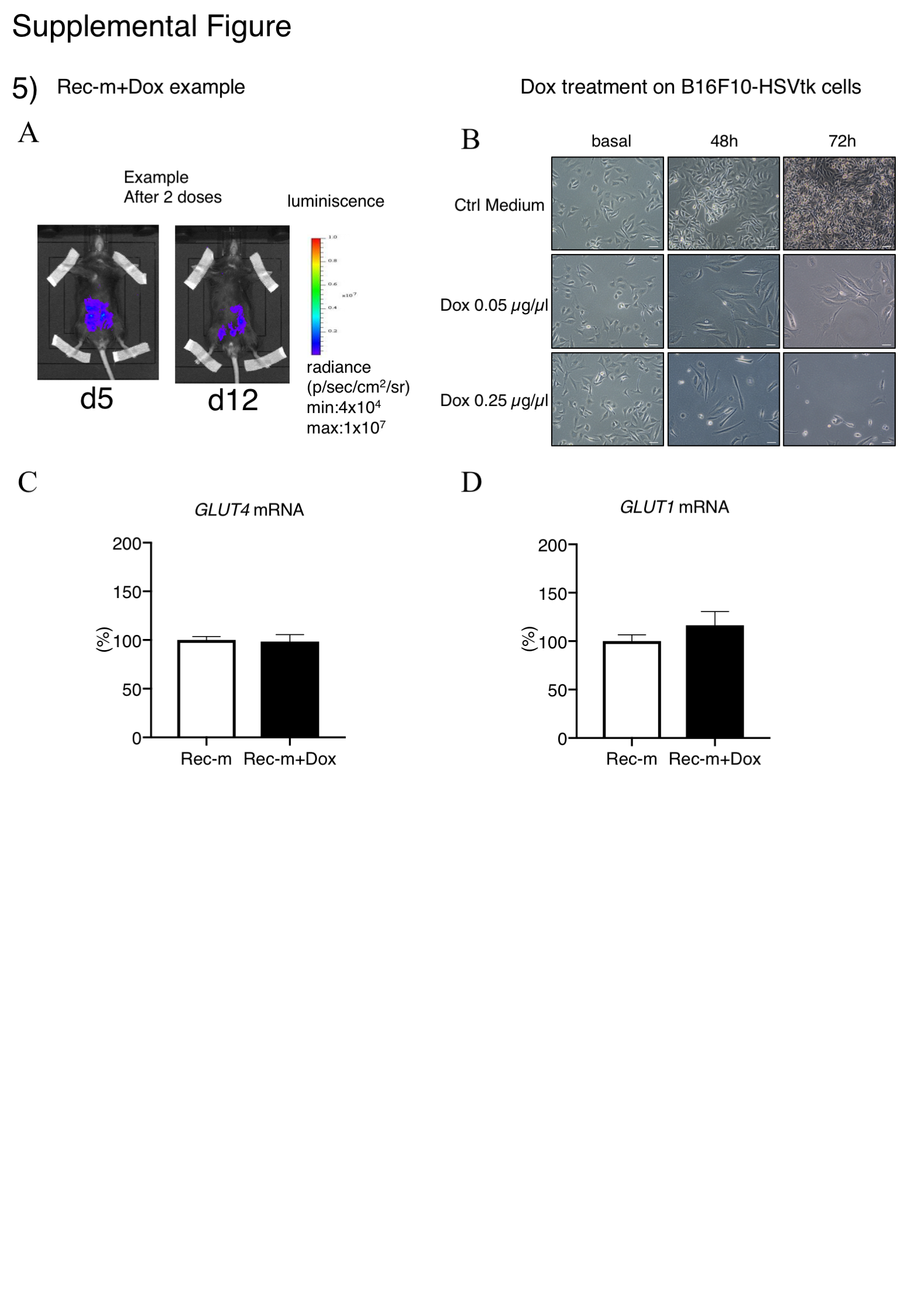

Supplement: Supplementary file 5 — Supplementary file5 Suppl. Fig. 5: Dox sensitivity of B16F10-HSVtk cells. A) Exemplary images of Dox treatment (after 2 doses at day 12 after tumor cell injection) monitored by IVIS. B) Representative images of Dox treated B16F10-HSVtk cells basal, 48h and 72h after Dox treatment, scale bar indicates 50 μm. Quantitative transcript levels determined by RNA-Seq. of C) GLUT4 and D) GLUT1 in LV tissue from n=2-4 individuals were pooled resulting in 3 pool samples per group (for: Rec-m, Rec-m+Dox). (TIFF 10263 KB) [file 395_2021_902_MOESM5_ESM.tiff]

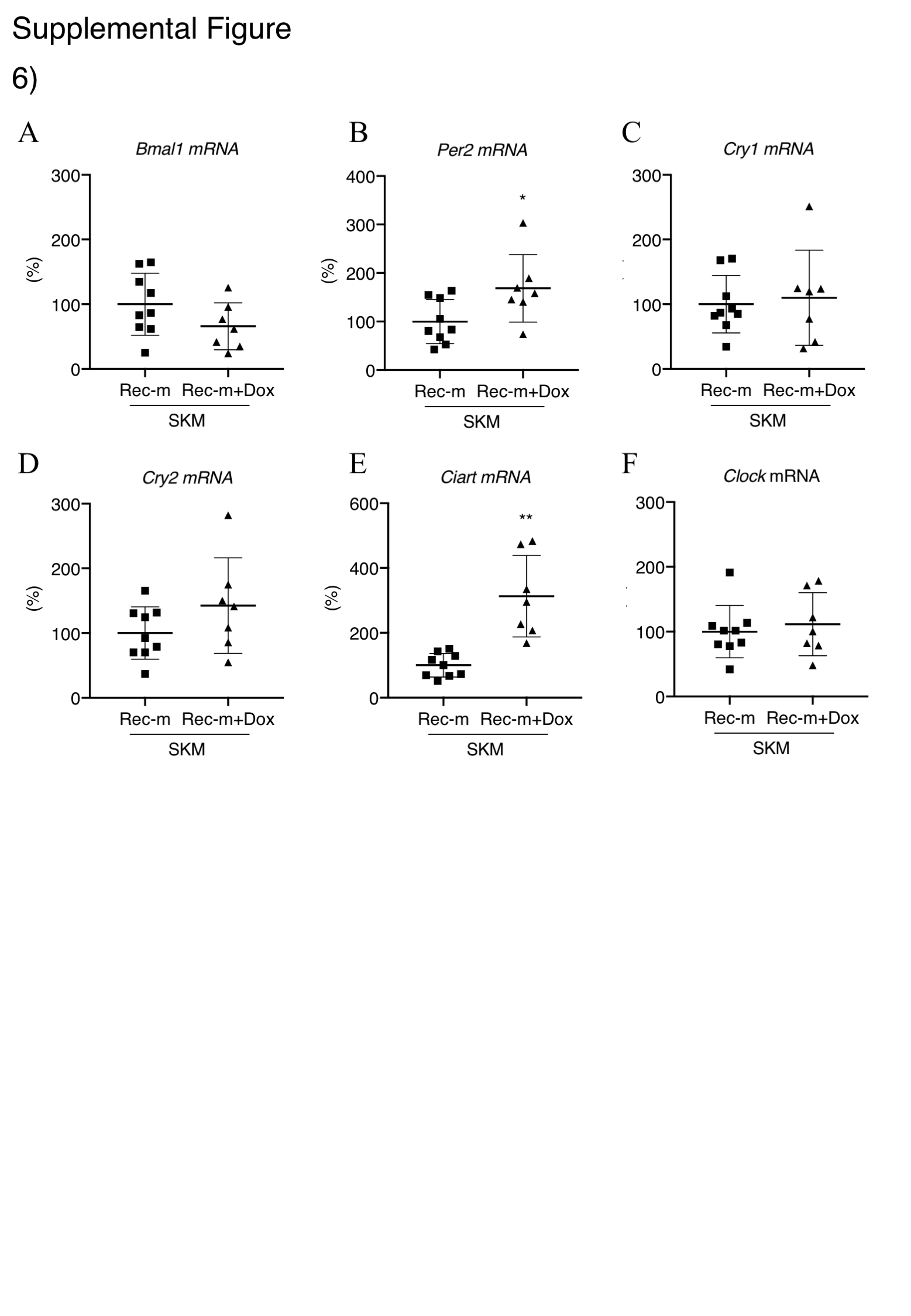

Supplement: Supplementary file 6 — Supplementary file6 Suppl. Fig. 6: Changes in skeletal muscle (SKM) RNA expression after recovery from cancer with and without Dox treatment. A-F) Quantitative mRNA levels (qRT-PCR) of A) Bmal1, B) Per2, C) Cry1, D) Cry2, E) Ciart and F) CLOCK in SKM tissue from Rec-m (n=9) and Rec-m+Dox (n=7) normalized to 18S. Data are depicted as mean ± SD, *P < 0.05, **P < 0.01 vs. respective Rec-m group, using 2-tailed-Student’s t-test. (TIFF 10263 KB) [file 395_2021_902_MOESM6_ESM.tiff]

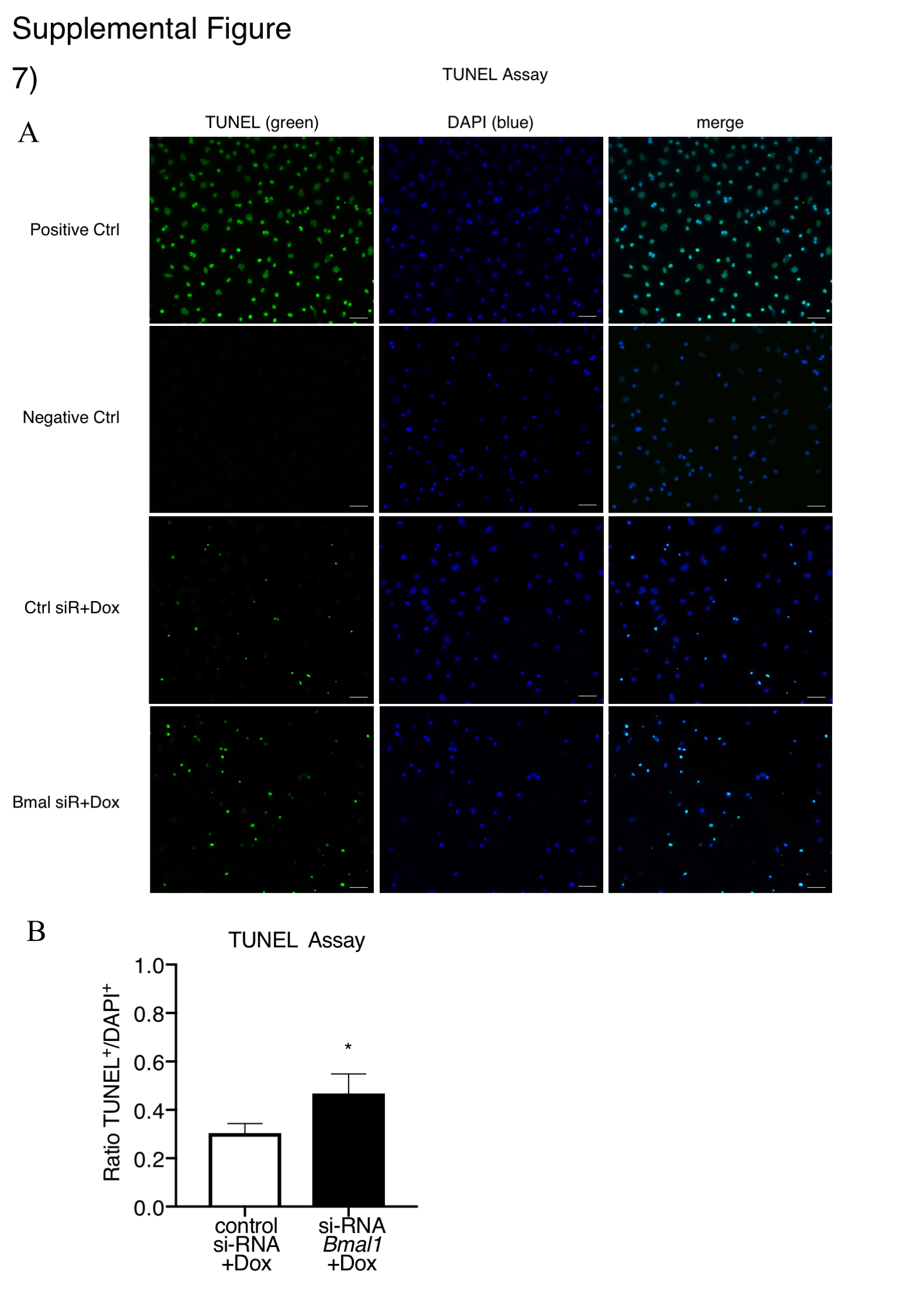

Supplement: Supplementary file 7 — Supplementary file7 Suppl. Fig. 7: Doxorubicin-induced increased apoptosis in NRCM with siRNA-mediated knockdown of BMAL1. A) Representative images of TUNEL+ (green), DAPI+ (blue) and merged TUNEL+/DAPI+ from neonatal rat cardiomyocytes treated with Dox for 24 hours after pre-treatment with Bmal1-siRNA or scrambled siR-control for 48 hours, scale bar indicates 50 μm. B) Bar graph depicts the ratio of TUNEL+ to DAPI+ NRCM treated with Dox for 24 hours after pre-treatment with Bmal1-si-RNA or scrambled siR-control for 48 hours from n=3 experiments. Data are depicted as mean ± SD, *P < 0.05 vs. scrambled siR-control using 2-tailed-Student’s t-test. (TIFF 10263 KB) [file 395_2021_902_MOESM7_ESM.tiff]

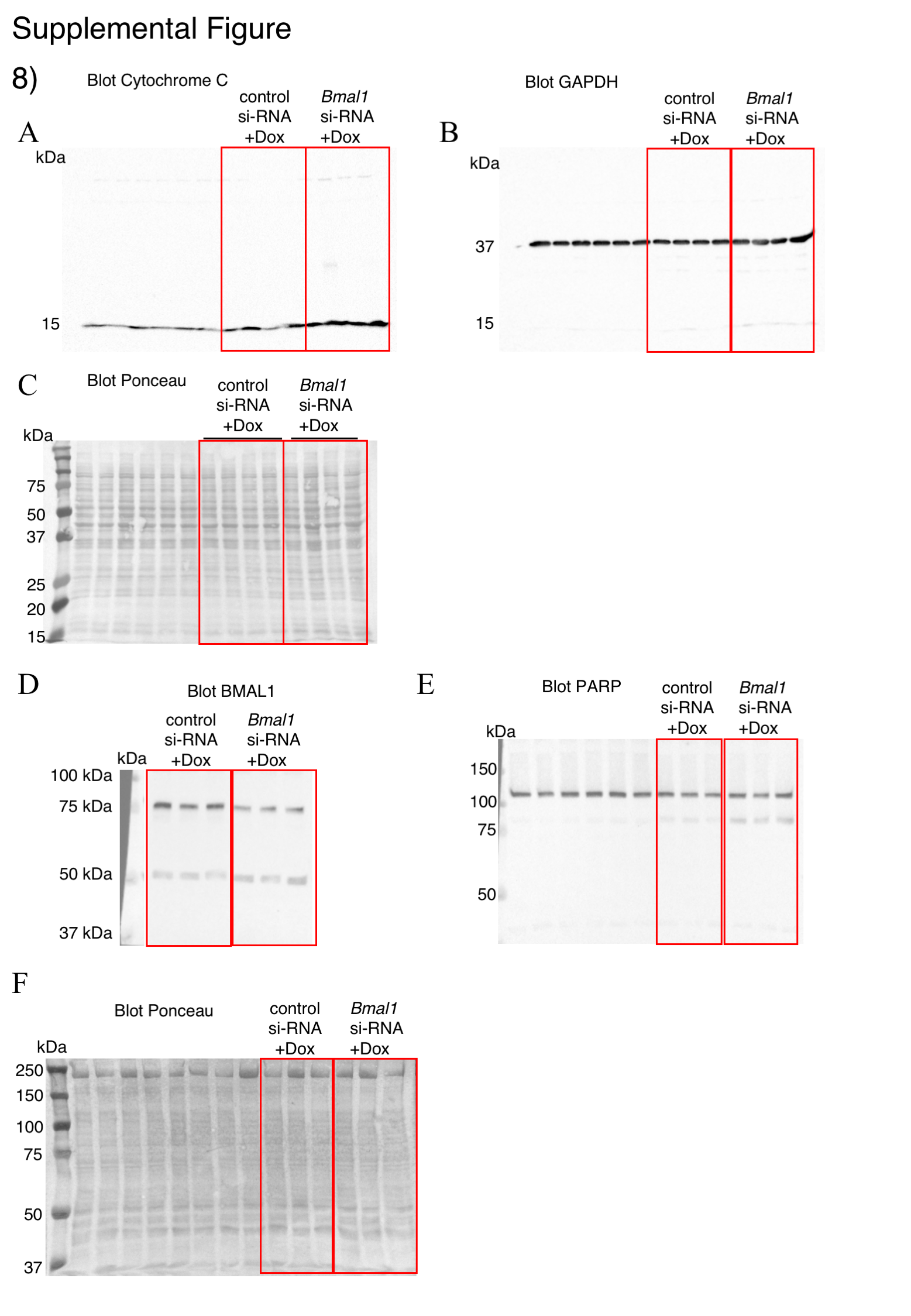

Supplement: Supplementary file 8 — Supplementary file8 Suppl. Fig. 8: Whole western blots of Figure 7. Whole gel images of representative western blots shown in Fig. 7C of A) cytochrome C, B) GAPDH from the cytosolic protein fraction of NRCM after treatment with Bmal1-si-RNA or scrambled siR-control for 48 hours followed by 24 hours Dox and corresponding quantification normalized on C) Ponceau staining, and in Fig. 7E of D) BMAL1, E) PARP, F) Ponceau, G) p-H2AX, H, I) caspase 3 and corresponding J) Ponceau staining from NRCM transfected with Bmal1-si-RNA or scrambled siR-control for 48 hours followed by 24 hours Dox. (TIFF 10263 KB) [file 395_2021_902_MOESM8_ESM.tiff]

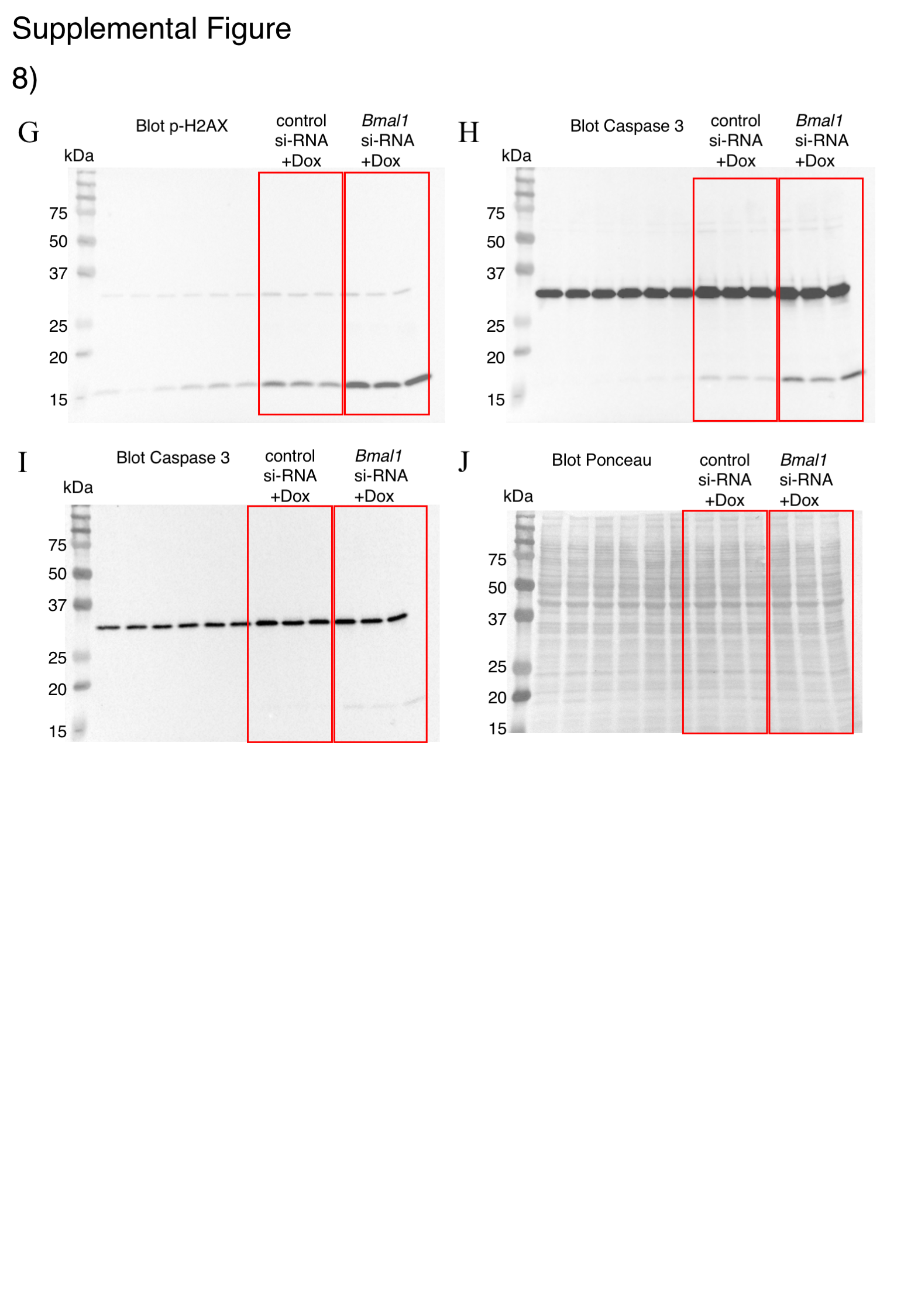

Supplement: Supplementary file 9 — Supplementary file9 (TIFF 10263 KB) [file 395_2021_902_MOESM9_ESM.tiff]
